# Spinal lumbar Urocortin 3-expressing neurons are associated with both scratching and Compound 48/80-induced sensations

**DOI:** 10.1097/j.pain.0000000000003435

**Published:** 2024-10-15

**Authors:** Marina C. M. Franck, Hannah M. Weman, Mikaela M. Ceder, Aikeremu Ahemaiti, Katharina Henriksson, Erica Bengtsson, Kajsa A. Magnusson, Harmen K. Koning, Caroline Öhman-Mägi, Malin C. Lagerström

**Affiliations:** a Department of Immunology, Genetics and Pathology, Uppsala University, Uppsala, Sweden; b Department of Materials Science and Engineering, Applied Materials Science, Uppsala University, Uppsala, Sweden

**Keywords:** Itch, Scratching, Dorsal horn, Spinal cord, Urocortin 3, Compound 48/80

## Abstract

Urocortin 3 is a neuropeptide that belongs to the corticotropin-releasing hormone family and is involved in mechanosensation and stress regulation. In this study, we show that *Urocortin 3* marks a population of excitatory neurons in the mouse spinal cord, divided into 2 nonoverlapping subpopulations expressing protein kinase C gamma or calretinin/calbindin 2, populations previously associated with mechanosensation. Electrophysiological experiments demonstrated that lumbar spinal Urocortin 3 neurons receive both glycinergic and GABAergic local tonic inhibition, and monosynaptic inputs from both Aβ and C fibers, which could be confirmed by retrograde trans-synaptic rabies tracing. Furthermore, *fos* analyses showed that subpopulations of lumbar *Urocortin 3* neurons are activated by artificial scratching or Compound 48/80-induced sensations. Chemogenetic activation of lumbar Urocortin 3-Cre neurons evoked a targeted biting/licking behavior towards the corresponding dermatome and chemogenetic inhibition decreased Compound 48/80-induced behavior. Hence, spinal lumbar Urocortin 3 neurons represent a mechanically associated population with roles in both scratching and Compound 48/80-induced sensations.

## Abstract 2

Supplemental Digital Content is Available in the Text.

Lumbar spinal Urocortin 3 neurons express markers associated with mechanosensation and subpopulations are activated by scratching or Compound 48/80. Inhibition reduced the response to Compound 48/80.

## 1. Introduction

Itch is evoked by the light mechanical stimulus of the skin or by chemical compounds, such as histamine, Compound 48/80,^58,61^ or chloroquine, which activate primary afferents and further involve distinct spinal dorsal horn circuits. Urocortin 3 (UCN3), or stresscopin, belongs to the corticotropin-releasing hormone (CRH) family and binds selectively to the corticotropin-releasing hormone receptor 2 (CRHR2).^23,35^ The UCN3/CRHR2 system has previously been associated with several physiological functions. For instance, hypothalamic Ucn3-expressing neurons are involved in risk assessment behavior,^22^ and intracerebroventricular injection of UCN3 activates the stress-related hypothalamic-pituitary-adrenal (HPA) axis in rats.^25^ In addition, UCN3 is linked to sensations such as mechanosensitivity of the gastric vagal afferents^36^ and protection against age- and noise-induced hearing loss.^14^ Moreover, spinal Ucn3-Cre neurons have been found to have a role in mechanical itch and alloknesis.^45^

In mice, the mRNA expression of *Ucn3* is detected in the small intestine, skin, hypothalamus, extended amygdala, and brainstem.^35^ In the mouse spinal cord, *Ucn3* is found exclusively in a few excitatory clusters of dorsal horn interneurons.^24,68^ Protein kinase C gamma (PKCγ) and calretinin/calbindin 2 (CALB2) are expressed in this area of the spinal cord in nonoverlapping populations.^47,48,52^ Both PKCγ and CALB2 populations are connected to the transmission of mechanical sensations and convey mechanical allodynia caused by distinct peripheral injuries, where PKCγ neurons are mainly associated with nerve injury persistent pain and CALB2 primarily with inflammatory pain.^47^ In addition, neuronal populations in the dorsal horn are central for chemical itch transmission,^3,42^ and peripherally delivered pruritogens induce *fos* expression in the dorsal horn.^17,65^

Herein, the restricted spinal dorsal horn expressional pattern of Ucn3-Cre prompted us to analyze the lumbar spinal *Ucn3*-expressing neurons, using chemogenetics, single-cell bioinformatics, retrograde trans-synaptic rabies virus tracing, electrophysiology, root stimulations, and stimulus-evoked *fos* analyses.

## 2. Material and methods

### 2.1. Animals

The Ucn3-Cre mouse line was created within the GENSAT project^20^ and purchased from the MMRRC (founder line KF31, US Davis, CA). Ucn3-Cre mice were crossed with C57BL/6J mice (Taconic, Denmark) or tdTomato (Gt(ROSA)26Sor^tm14(CAG-tdTomato)Hze^; Allen Brain Institute) mice to identify Ucn3-Cre(+) neurons.

Protocols related to the animals used in this study were approved by the local animal research ethical committee (Uppsala djurförsöksetiska nämnd) and followed the Swedish Animal Welfare Act (Svensk författningssamling [SFS] 2018:1192), The Swedish Animal Welfare Ordinance (SFS 2019:66) and the Regulations and General Advice for Laboratory Animals (SJVFS 2019:9, Saknr L 150), according to permit numbers C58/14, C135/14, C110/16, 5.8.18-11551/2019, 5.8.18-01217/2019, 5.8.18-19421/2019, 5.8.18-01503/2023, 5.8.18-01428/2023. The Ucn3-Cre allele was kept heterozygous and both female and male mice were used if not otherwise indicated (please see each paragraph).

### 2.2. Genotyping by polymerase chain reaction

Tissue biopsies from ear marking taken at the age of weaning were incubated in 50 to 100 µL of buffer consisting of 25 mM NaOH and 200 µM EDTA, in a shaking block (BIOER, Mixing Block MB-102, Hangzhou, China, 300 speed) at 96°C for 25 minutes and placed on ice before the sample was neutralized with 50 to 100 µL of Tris-HCl (40 mM), pH 8.0. Genotyping primers used: *Cre*: *5′-ACG​AGT​GAT​GAG​GTT​CGC​AAG​A* (forward, mutant allele) and *5′-ACC​GAC​GAT​GAA​GCA​TGT​TTA​G* (reverse, mutant allele); *tdTomato*: *5′-CTG​TTC​CTG​TAC​GGC​ATG​G* (forward, mutant allele)*, 5′-GGC​ATT​AAA​GCA​GCG​TAT​CC* (reverse, mutant allele), *5′-AAG​GGA​GCT​GCA​GTG​GAG​TA* (forward, wild type allele), and *5′-CCG​AAA​ATC​TGT​GGG​AAG​TC* (reverse, wild type allele).

### 2.3. Immunohistochemical analyses

Mice were anesthetized with an intraperitoneal injection of a 1:1 (volume) mixture of medetomidine (Domitor, 1 mg/mL, Orion Pharma, Stockholm, Sweden) and Ketamine (Ketalar, 10 mg/mL, Pfizer, Stockholm, Sweden), and tissue was fixed by transcardial perfusion through the left ventricle with phosphate-buffered saline (PBS) followed by 4% formaldehyde (FA) (Histolab, Gothenburg, Sweden) in PBS. Tissues of interest (spinal cord, brain, and/or dorsal root ganglia [DRG]) were excised and immersed in 4% FA in PBS overnight. The tissues were later washed with PBS, equilibrated in a sucrose series (Sigma-Aldrich, 10%-30% sucrose in PBS), and frozen in optimal cutting temperature (OCT) compound (Bio-Optica, Milano, Italy). Tissue sections of the spinal cord and DRG were cut on a cryostat (Leica Cryocut 1800, Berlin, Germany) at 14 to 20 µm. Brain sections were cut on a vibratome (Leica VT1000S, Leica) at 70 µm.

For immunohistochemical analysis of frozen sections, slides were thawed and dried for 30 to 45 minutes and then washed in PBS 3 times for 10 minutes. Sections were blocked for 1 hour in blocking solution (0.25% gelatin (Fisher Scientific, Gothenburg, Sweden), 0.5% Triton X-100 (Sigma-Aldrich, Stockholm, Sweden) in PBS) at room temperature. Primary antibodies were incubated on the slides for approximately 16 hours at 4°C in blocking solution. Sections were washed 3 times for 15 minutes in PBS and then incubated with secondary antibodies in blocking solution (with DAPI (4',6-diamidino-2-phenylindole) 200 ng/mL, VWR, Stockholm (Spånga), Cat# 40011) for 2 hours at room temperature. Sections were washed 3 times for 15 minutes in PBS and then mounted in ProLong Gold Antifade Mountant (Life Technologies, Stockholm, Sweden) or Fluoroshield (Abcam, Cambridge, United Kingdom).

Primary antibodies: Rabbit anti-protein kinase C gamma (PKCγ, Santa Cruz Biotechnology, Dallas, TX, Cat# sc-211, RRID:AB_632234, used at 1:500), mouse anti-calretinin (CALB2, Swant, Bern, Switzerland Cat# 6B3, used at 1:500), sheep anti-EBF transcription factor 2 (EBF2, ThermoFisher Scientific, Stockholm, Sweden, Cat# PA5-47890, RRID: AB_2577232, used at 1:500), rabbit anti-paired box 2 (PAX2, Covance/Biolegend, Stockholm, Sweden, Cat# poly19010, used at 1:500), mouse anti-neuronal nuclei (NEUN, Millipore, Darmstadt, Germany Cat# MAB377, used at 1:200), guinea pig anti-substance P (SP, Abcam, Cat# ab10353, used at 1:1000), chicken anti-green fluorescent protein (GFP, Aves Labs, Davis, CA, Cat# GFP-1020, RRID: AB_2307313, used at 1:1000), rabbit anti-neurofilament 200 (NF200, Sigma-Aldrich, Cat# N4142, RRID:AB_477272, used at 1:1000), rabbit anti-parvalbumin (PARV, Swant, Cat# PV27, RRID:AB_2631173, used at 1:1000), rabbit anti-tyrosine hydroxylase (TH, Millipore, Cat# AB152, RRID:AB_390204, used at 1:1000), rabbit anti-tropomyosin receptor kinase A (TRKA, Abcam, Cat# ab8871, used at 1:3000), rabbit anti-calcitonin gene-related peptide (CGRP, Peninsula Laboratories, San Carlos, CA, Cat# T-4239.0050, RRID:AB_518150, used at 1:500), and isolectin GS-IB4-Alexa647 (IB4, Invitrogen, Stockholm, Sweden Cat# I32450, used at 1:1000). Secondary antibodies: donkey anti-chicken Alexa488 (Cat# A78948), donkey anti-mouse Alexa647 (Cat# A-31571), donkey anti-mouse Alexa488 (Cat# R37114), donkey anti-mouse Alexa594 (Cat# R37115), donkey anti-rabbit Alexa647 (Cat# A-31573), donkey anti-rabbit Alexa488 (Cat# R37118), and donkey anti-sheep Alexa647 (Cat# A-21448) were from ThermoFisher Scientific. Donkey anti-guinea pig Alexa488 (Cat# 706-545-148) was from Jackson ImmunoResearch, Cambridge, United Kingdom. The following sections were excluded during analysis due to poor signal of immunohistochemistry: NF200 1 section and TH 1 section. The following tissue sections were excluded during the analysis due to poor tissue quality: rabies tracing of spinal cord, GFP and Pax2 staining: 5 sections.

### 2.4. Viral injection

Adult mice were initially anesthetized in a 4% isoflurane (FORANE, Baxter, Apoteket, Stockholm, Sweden) containing box, and when fully anesthetized, the mice were moved to a stereotaxic frame with a breathing mask where the isoflurane concentration was kept at 1.5% to 2% throughout the procedure. To prevent eye damage, Oftagel was applied (Santen Oy, Tammerfors, Finland), and the body temperature was monitored and kept at 35 to 37°C using a heating pad (CMA, Kista, Sweden). At the injection site, the mice were administered subcutaneously with bupivacaine (Marcain, 2 mg/kg, AstraZeneca, London, United Kingdom), and for postsurgery analgesia, the mice were administered subcutaneously with karprofen (Norocarp vet, 5 mg/kg, N-vet, Uppsala, Sweden or Rimadyl Bovis vet, 4 mg/kg, Zoetis Animal Health, Copenhagen, Denmark). If the mouse received 4 mg/kg Rimadyl Bovis vet, Buprenorfin (Vetergesic Vet, Orion Pharma, Espoo, Finland, 0.05 mg/kg) was subcutaneously administered before the mouse woke up. Within 24 hours postsurgery, the mice were again administered 4 to 5 mg/kg karprofen for postsurgery analgesia. After analgesic injection, the dorsal skin was shaved and cleaned with sterile saline (0.9% NaCl, B. Braun Medical AB, Stockholm, Sweden) and chlorhexidine (Fresenius Kabi, Bad Homburg, Germany), where after a 1-cm incision was made in the skin to expose the T13 and L1 vertebrae. Sterile saline was continuously added to the tissue to keep it moist throughout the procedure. The connective tissue was gently separated along these vertebrae, and a clamp was inserted ventrally of the L1 transverse process for stabilization of the spine; when stabilized, the ligaments connecting T13 and L1 were separated to expose the spinal cord. Thereafter, 500 nL of *AAV8.hSyn-DIO-hM3D(Gq)-mCherry*^30^[pAAV-hSyn-DIO-hM3D(Gq)-mCherry, kindly donated by Bryan Roth (Addgene, Watertown, MA, viral prep # 44361-AAV8; http://n2t.net/addgene:44361; RRID:Addgene_44361), lot#: 43877, with following titers: titer 1: 2.0 × 10^13^ GC/mL; lot # v27924, titer 2: 2.2 × 10^13^ GC/mL; lot #v78582, titer 3: 2.1 × 10^13^ GC/mL (Addgene)]; *AAV8.hSyn-DIO-mCherry* [(lot# AV4981F, titer: 6.1 × 10^12^ GC/mL) from UNC vector core, Chapel Hill, NC or Addgene (pAAV-hSyn-DIO-mCherry, kindly donated by Bryan Roth (Addgene viral prep # 50459-AAV8; http://n2t.net/addgene:50459; RRID:Addgene_50459), lot#: v61605, titer: 2.2 × 10^13^ GC/mL)] was injected at 2 sites in the right dorsal horn (RC: 0.8 mm apart, ML: 0.35-0.4 mm, DV: 0.4-0.5 mm) with the eye of the needle pointing laterally. *AAVDJ.EF1a-DIO-HTB* [pAAV-Ef1a-DIO-HTB was a gift from Edward Callaway (Addgene plasmid # 44187; http://n2t.net/addgene:44187; RRID:Addgene_44187), lot date: 20/12-2018, titer: 1.09 × 10^12^ vg/mL) produced by the Salk Institute, San Diego, CA, GT3 (Gene Transfer, Targeting, and Therapeutics) core facility and provided by Salk investigator John Naughton with funding from NIH-NCI CCSG: P30 014195, an NINDS R24 Core Grant and funding from NEI] was injected into the L4-L5 (L5-L6 for HTB virus) spinal dorsal horn (as caudal as possible from zeroed midline, ML: 0.4 mm, DV: 0.4 mm, with needle eye pointing rostrally) using a 10 µL Nanofil Hamilton syringe (World Precision Instruments, Friedberg, Germany) with a 34 g beveled needle (World Precision Instruments) monitored by a micro syringe pump controller (World Precision Instruments) at 50 nL/minute. For injections of *AAV8.hSyn-DIO-hM4D(Gi)-mCherry*^30^ [pAAV-hSyn-DIO-hM4D(Gi)-mCherry, kindly donated by Bryan Roth (Addgene viral prep # 44362-AAV8; http://n2t.net/addgene:44362; RRID:Addgene_44362), lot#: v86749, titer: 1.8 × 10^13^ GC/mL) from Addgene, titer: 1.8 × 10^13^ GC/mL) from Addgene], the virus was injected at 2 sites in the right dorsal horn (RC: 0 or −0.5 mm, ML: 0.3 mm, DV: 0.6 mm) with the eye of the needle pointing laterally. For rabies virus retrograde trans-synaptic tracing, 1 or 2 injections of 500 nL of AAV2.EF1a-FLEX-GFP-TVA (titer: 4.44 × 10^11^ vg/mL, lot# A021404b, Addgene plasmid # 26198; http://n2t.net/addgene:26198; RRID:Addgene_26198^62^) were performed unilaterally between vertebrae T13 and L1, injection coordinates RC: 0 to 0.9 mm apart, ML: 0.35 to 0.5 mm, DV: 0.5 mm from surface. Furthermore, 1 to 2 weeks later, 1 or 2 injections were performed in the same locations with a mixture of 25 nL AAV9-CAG-FLEX-oG-WPRE-SV40-pA (titer: 1.78 × 10^11^ vg/mL, Addgene plasmid # 74292; http://n2t.net/addgene:74292; RRID:Addgene_74292^28^) and 475 nL of EnvA-ΔG-mCherry rabies virus (titer: 1.41 × 10^8^ genome copies/mL, Addgene plasmid # 32636; http://n2t.net/addgene:32636; RRID:Addgene_32636^44^). All viruses for rabies virus retrograde trans-synaptic tracing were purchased from Salk Institute, kindly provided by Edward Callaway and Salk investigator John Naughton. To prevent leakage and withdrawal of the virus, the needle was left in the injection site for 5 minutes after the finished injection. When the injection was completed, the spine was detached from the clamp. The connective tissue and skin were sutured and cleaned with sterile saline before the mouse was placed on a heating pad in the cage to emerge from anesthesia. The mice were subjected to behavioral experiments or sacrificed for tissue analyses after a minimum of 1 week to allow sufficient expression of viral genes. The spinal cords from all mice analyzed in behavioral experiments were examined for mCherry to secure sufficient expression of viral genes in the correct segments. Mice without apparent mCherry expression were excluded from the analysis.

### 2.5. Tissue preparation for fluorescent in situ hybridization

*Virally injected Ucn3-Cre mice:* when 14 days had passed since the AAVDJ.EF1a-DIO-HTB bilateral L5/L6 injection, the mice (3 males, 9-12 weeks old when perfused) were injected intraperitoneally with 0.6 to 0.7 mL Ketamine (Ketalar, 10 mg/mL, Pfizer, Strängnäs, Sweden) and medetomidine (Domitor, 1 mg/mL, Orion Pharma) (1:1). *Ucn3-Cre;tdTomato mice*: Mice were administered intraperitoneally with 0.2-0.3 mL Ketamine (Ketalar, 50 mg/mL, Pfizer) and medetomidine (Domitor, 1 mg/mL, Orion Pharma) (1:1). *Sensory stimulated C57BL/6J mice*: 40 minutes after sensory stimulation, the mice were administered intraperitoneally with 0.1 mL (for the urethane experiment) or 0.7 to 0.8 mL (for Hargreaves stimulation) of Ketamine (Ketalar, 10 mg/mL, Pfizer) and medetomidine (Domitor, 1 mg/mL, Orion Pharma) (1:1). After the anesthesia, mice were sacrificed in the same manner; to minimize mRNA degradation, the mice were perfused with ice-cold and autoclaved PBS. In the same solution, the spinal cords were quickly dissected, where the lumbar area around the injection sites was isolated and embedded in an OCT medium (Bio-Optica, Italy). Immediately after the embedding, the tissues were snap-frozen on dry ice in −80°C isopentane (Sigma-Aldrich), at which temperature the tissues were stored until sectioning. The spinal cords were cryosectioned (Leica Cryocut 1800) into 12 to 14 µm sections and were collected onto Superfrost Plus (Thermo Scientific) glass slides as a series consisting of 6 slides with 7 to 8 sections/slide for the Ucn3-Cre AAVDJ.EF1a-DIO-HTB injected mice, 6 slides with 10 sections/slide for the Ucn3-Cre;tdTomato mice and 8 slides with 3 sections/slide for the sensory stimulated mice. To minimize contamination and mRNA degradation, the completed series were stored at −21°C; after sectioning was completed, the slides were stored at −80°C until fluorescent in situ hybridization protocol commenced.

### 2.6. Fluorescent in situ hybridization

*Virally injected Ucn3-Cre and sensory stimulated C57BL/6J mice:* The fluorescent in situ hybridization (FISH) was performed using the RNAscope Fluorescent Multiplex kit for fresh frozen tissue (Advanced Cell Diagnostics (ACD), Newark, CA, Cat# 320850) with minor modifications.^63^ Sections containing Ucn3-Cre.HTB cells or spinal L5-L6 sections from the sensory stimulated C57BL/6J mice were taken from −80°C and placed in room temperature 4% FA in PBS (Histolab) for 15 minutes before 2 minutes wash in room temperature autoclaved PBS. Thereafter, the slides were dehydrated in a stepwise concentration increase of EtOH: 3 minutes in 50%, 3 minutes in 70%, and 2 times for 5 minutes in 100% EtOH (Merck KGaA, Darmstadt, Germany). The slides were placed at room temperature for 5 minutes to air dry, where after a hydrophobic barrier was made around the area of interest (*Ucn3-Cre.HTB*: 3-5 sections/mouse, total 12 sections; *sensory stimulation*: 3 sections/mouse, total 9 sections/stimulation, except for pinch where 17 sections were used) using an ImmEdge pen (Vector Laboratories, Newark, CA,). The dried sections were thereafter incubated in Protease IV for 40 minutes at room temperature followed by washing 3 times for 5 minutes in room temperature autoclaved PBS, before being incubated with *Ucn3* and *fos* probes (Ucn3-Mm-C3, Cat# 464861-C3, 1:50; fos-Mm-C1, Cat# 316921) for 2 hours at 40°C in a hybridization oven (HybEZII Oven, ACD). In the *Ucn3-Cre.HTB* analysis, only the *Ucn3* probe was incubated. The subsequent amplification steps were performed at 40°C in the hybridization oven, and the sections were washed 2 times for 2 minutes in room temperature washing buffer between each amplification step: AMP 1-FL for 30 minutes, AMP 2-FL for 15 minutes, AMP 3-FL for 30 minutes and AMP 4-FL for 15 minutes. For Ucn3-Cre.HTB sections, the coloring step using AMP 4-FL was performed to enable the combination with the viral fluorescence. Last, the slides were washed 2 times for 2 minutes in washing buffer before 30 seconds incubation in DAPI and mounting in Anti-Fade Fluorescence Mounting Medium (Abcam). The slides were covered with glass slides (Menzel-Gläser, Braunschweig, Germany) and were left at 4°C to dry. The slides were stored at this temperature until imaging. One section used for in situ hybridization was excluded during postanalyses due to poor in situ staining. *Ucn3-Cre;tdTomato mice:* The fluorescent in situ hybridization (FISH) was performed using the RNAscope Fluorescent Multiplex kit for fresh frozen tissue (ACD, Cat# 323110) with minor modifications.^63^ Spinal L5-L6 sections from *Ucn3-*Cre*;tdTomato* mice were taken from −80°C and fixated in 4% FA in PBS, washed, and dehydrated in EtOH as described above. The slides were thereafter placed at room temperature for 5 minutes to air dry and a hydrophobic barrier was made around the area of interest (*Ucn3-Cre;tdTomato*: 3 sections/mouse (males), total 6 sections and 3 control sections) using an ImmEdge pen (Vector Laboratories). The dried sections were thereafter incubated in Protease IV (ACD, Cat# 322336) for 40 minutes at room temperature followed by washing 3 times for 5 minutes in room temperature autoclaved PBS, before being incubated with *Ucn3* and *tdTomato* probes (Ucn3-Mm-C3, Cat# 464861-C3, 1:50; tdTomato-Mm-C2, Cat# 317041-C2, 1:50) for 2 hours at 40°C in a hybridization oven (HybEZII Oven, ACD). The subsequent amplification and color development steps were performed at 40°C in the hybridization oven, and the sections were washed 2 times for 2 minutes in room temperature washing buffer between each step: AMP 1-FL for 30 minutes, AMP 2-FL for 30 minutes, and AMP 3-FL for 15 minutes. To develop a color signal, HRP-C2 or HRP-C3 was incubated for 15 minutes, followed by 30-minute incubation of the chosen fluorophore: TSA Vivid 520 (ACD, Cat# 7534) or TSA Vivid 650 (ACD, Cat# 7536). Fluorophores were diluted 1:5000 in TSA Buffer (ACD; Cat# 322809). Thereafter, HRP was blocked with an HRP blocker for 15 minutes, and the color development steps were repeated for the second probe. Last, the slides were washed 2 times for 2 minutes in washing buffer before 30 seconds incubation in DAPI and mounting in Anti-Fade Fluorescence Mounting Medium (Abcam). The slides were covered with glass slides (Menzel-Gläser, Germany) and were left at 4°C to dry. The slides were stored at this temperature until imaging. No sections used for in situ hybridization were excluded during postanalyses.

### 2.7. Image acquisition and quantification

Images of RNAscope and immunofluorescence treated sections were acquired using a wide field Olympus BX61WI fluorescent microscope (Olympus, Tokyo, Japan) or an Axio Imager.Z2 (Zeiss, Oberkochen, Germany) with 10x or 20x magnification, where each channel was set to be automatically optimized for each image but had to be further optimized during image analysis. In this study, the optimal intensity and contrast were set for 1 image (reference image), and the settings of the other images were set to match the reference image. The images were manually quantified using the Fiji (ImageJ, 1.52f) Cell Counter plugin, where 1 read of the targeted gene was visualized as 1 dot. *Ucn3-Cre.HTB and Ucn3-Cre;tdTomato*: all Ucn3-Cre.HTB or Ucn3-Cre;tdTomato cells with DAPI overlap were considered cells and were considered to express *Ucn3 or tdTomato* if the overlapping # dots ≥ 5 (HTB n mice: 3 males, n sections/mouse: 3-5, 12 sections in total; *tdTomato* n mice: 2 males, n sections/mouse 3, 6 sections in total, 1 control (tdTomato(+)Ucn3-Cre(−), n sections/mouse 3). The data were presented as the mean proportion of Ucn3-Cre.HTB or Ucn3-Cre;tdTomato cells±SEM. *Sensory stimulated C57BL/6J*: a DAPI cell was considered to express the targeted gene (*Ucn3* and *fos*) if the # dots ≥ 5 for *Ucn3* and # dots ≥ 5 for *fos* (n mice: 2 females and 1 male or 1 female and 2 males/stimulus, 3 sections/mouse, except for pinch where 5-6 sections/mouse were used). The representative images in the figures were chosen from the same mouse, and to the greatest extent possible, the dorsal horn was from the same section. However, if the contralateral side was damaged, a section close to the representative ipsilateral image from the same sectioning series was chosen. The observer was blind to the sensory treatment applied while doing the quantifications. For size-frequency analysis of TRKA(+) neurons, the neurons were manually outlined in ImageJ and their area was measured. Only cells with a visible nucleus were included. All images were transformed to colorblindness-friendly pseudocolor using ImageJ. Red fluorescence (Alexa555) was presented as magenta, green (Alexa488) as yellow, and far red (Alexa647) as cyan.

### 2.8. Preprocessing of Zeisel et al. spinal cord single-cell mRNA sequencing dataset for Urocortin 3 expression analysis

To molecularly examine the spinal neurons expressing *Ucn3* and validate the immunohistological findings, the expression of targeted genes was investigated in *Ucn3*-expressing cells from the Zeisel et al. spinal cord dataset^68^ similar as described in Reference 16. The “l6_r3_spinal_cord_neurons.loom” file contains expression data of 27,998 genes in 1922 single-cells from Vgat-Cre:tdTomato with mixed CD-1 and C57BL/6J background,^68^ obtained by using the 10x Genomics method. The dataset was acquired from http://linnarssonlab.org/ and analyzed using SCANPY 1.9.1^66^ in Python 3.8.8. The full code can be found at (https://github.com/HannahMWeman/ucn3-analysis-zeisel-et-al-spinal-cord). First, the neurons annotated by Zeisel et al. (2018) to be true spinal cord neurons were isolated, resulting in 1744 neurons and 27,998 genes. For gene filtering, all genes that were expressed in less than 3 cells (SCANPY, sc.pp.filter_genes) and all cells expressing less than 200 genes (SCANPY, sc.pp.filter_cells) were excluded. The metrics of general gene expression and mitochondrial genes were calculated (SCANPY, pp.calculate_gc_metrics),^39^ and the distributions of the calculated metrics were visualized (SCANPY, pl.violin; Seaborn, jointplot). The neurons with less than 4% mitochondrial gene reads (SCANPY, pct_counts_mt < 4), high total counts (SCANPY, “log1p_total_counts” > 6.5), and distributed gene counts and broad gene capture (SCANPY, “logp_n_genes_by_counts” > 6.0, “pct_counts_in_top_50_genes” < 50) were isolated. The dataset did not contain ERCC genes since the 10x Genomics method, which was used by Zeisel et al.,^68^ does not include ERCC sequences, and thus cells were not filtered based on the expression criteria of these sequences. The inclusion criteria resulted in 1707 spinal cord neurons and 14,642 genes to be used for the expressional analysis. Finally, the counts per cell were thereafter normalized to the medium number of counts (SCANPY, pp.normalize_per_cell) followed by dataset normalization (SCANPY, pp.log1p).

### 2.9. Single-cell mRNA sequencing analysis of Urocortin 3-expressing cells

First, the expression of *Ucn3* was visualized in the individual Zeisel et al.^68^ annotated spinal cord cell types (SCANPY, pl.violin). From the visualization, it was observed that *Ucn3* expression was exclusively present in the excitatory SCGLU7 and SCGLU9 clusters. To investigate the expressional similarities of these clusters, all excitatory spinal cord neurons were isolated and the topography was computed based on the most highly expressed genes (SCANPY, pp.pca^46^; pp.highly_variable_genes)^54,59,69^ and thereafter visualized with uniform manifold approximation and projection (UMAP) graph (SCANPY, pp.neighbors (n_neighbors= 10, n_pcs = 10); tl.umap; pl.umap)).^40^ To further investigate if there were expressional differences between the *Ucn3*-expressing neurons in the SCGLU7 and SCGLU9 clusters (*Ucn3*-SCGLU7 and *Ucn3*-SCGLU9, *Ucn3* considered expressed if log1*P* > 0.5), the z-scores and adjusted *P*-values (*t* test, Benjamini-Hochberg for adjusting the false discovery rate (FDR)) were calculated and ranked for the top differentially expressed genes (SCANPY, tl.rank_genes_groups). The expression for the top 40 differentially expressed genes from each *Ucn3* cluster was visualized with a dot plot (SCANPY, pl.rank_genes_groups_dotplot). Among the top differentially expressed genes, *Calb2* (encodes CALB2) was found in SCGLU7 and *Prkcg* (encodes PKCγ) was found in SCGLU9; therefore, the prevalence of these genes in the *Ucn3*-SCGLU7 and *Ucn3*-SCGLU9 subclusters was calculated (gene considered expressed if log1 *P* > 0.5).

### 2.10. Single-cell mRNA sequencing analysis of Calb2 expression in *Prkcg*-expressing cells

The expression of *Calb2* was investigated in *Prkcg*-expressing cells in the Zeisel et al. dataset.^68^ The level of *Calb2* expression was visualized (SCANPY, pl.violin) in low *Prkcg*-expressing (0.1 > log1 *P* > 1.0) and high *Prkcg*-expressing (log1 *P* > 1.0) cells, respectively. The occurrence of *Calb2* expression (gene considered expressed if log1 *P* > 0.1) was calculated for low- or high-expressing *Prkcg* cells.

### 2.11. Electrophysiological recording

Spinal cord transverse slices were made according to a preparation procedure previously described.^16^ For root stimulation, the spinal cord was cut into 400 µm transverse slices at a 60° angle. After incubation, the slice was transferred to a recording chamber for electrophysiological analysis. Ucn3-Cre;tdTomato neurons were visualized on a Prime BSI Express scientific sCMOS camera (Teledyne Photometrics, Tucson, AZ) using a 60x or 20x water-immersion objective (LUMPlan FI, 0.90 numerical aperture (NA), Olympus), which was connected to a green (550 nm) fluorescent LED light source (CoolLED system, Andover, United Kingdom). Patch electrodes (6-10 MΩ) were pulled from borosilicate glass capillaries (GC150F-10 Harvard Apparatus, Holliston, MA) with a flaming/brown micropipette puller (P-1000, Shutter Instrument, Novato, CA). The pipette was filled with an internal solution containing the following (in mM): 130 K-gluconate, 40 HEPES, 1.02 MgCl_2_, 2.17 MgATP, and 0.34 NaGTP, with pH adjusted to 7.2 using 1 M KOH. Liquid junction potential was corrected before each patched neuron. Whole-cell patch-clamp signals were amplified with a MultiClamp 700B amplifier (Molecular Devices, San Jose, CA), digitalized at 20 kHz with Digidata 1440A (Molecular Devices), low pass filtered at 10 kHz, and acquired in WinWCP software (Dr. J. Dempster, University of Strathclyde, Glasgow, United Kingdom). The dorsal root was stimulated with an A365 Stimulus Isolator (World Precision Instruments) using a suction pipette. Stimulation pulses with a duration of 0.1 milliseconds were used for Aα/β fiber activation, while 0.1 milliseconds and 0.5 milliseconds of pulse durations were used for C fiber activation. The monosynaptic inputs were determined with transduction velocities of different afferent fibers^45^ and nonfailure responses with consistent onset latencies, where a patched cell responded to a minimum of 10 consecutive root stimulations at 1 Hz and the latency variation was less than 1 millisecond.^45,51^ In addition, to reveal tonic inhibitory currents, the glycine receptor antagonist strychnine (4 µM, Sigma-Aldrich) and the GABA receptor antagonist bicuculline (20 µM, Sigma-Aldrich) were applied to the recording chamber to block glycine and GABA receptor activities, respectively. Data analysis was done by Clampfit 10.3 (Molecular Devices), Mini Analysis 6 (Synaptosoft, Informer Technologies, Inc., LA, CA), and GraphPad Prism 6 (GraphPad Software, Boston, MA).

### 2.12. Sensory stimulation in urethane anesthetized mice for fos detection

The *fos* analysis was performed as previously described.^64^ Adult C57BL/6J mice (3 mice of mixed sex/stimulus, 15 mice in total, 10-14 weeks old) were initially anesthetized with 2 g/kg urethane (Sigma-Aldrich, 125 mg/mL in saline) through intraperitoneal injection, and when the mouse had been fully anesthetized for 10 minutes, the mouse was subjected to the stimulus. To prevent eye damage and dehydration, Oftagel (Santen Oy) was applied to both eyes, and the mouse was injected subcutaneously with 0.5 mL saline. To maintain body temperature, a glove filled with body temperature water was placed next to the mouse and was continuously replaced to sustain temperature. The mouse was injected with 10 µL sterile saline (1 female and 2 males) or 20 µg Compound 48/80 (Sigma-Aldrich, Cat# C2313, dissolved in sterile saline, 1 female and 2 males) subcutaneously in the right dorsolateral calf. We chose to place the injections subcutaneously to increase reproducibility and reduce the risk of injection-induced injuries to the skin. For mechanical stimulation, the mouse was either subjected to scratching (2 females and 1 male) or pinching (1 female and 2 males) of the skin in the right dorsolateral calf. The scratching was performed for 30 seconds with 2 Hz similar to a study by Davidson et al.^10^ but with a lighter pressure of approximately 300 mN (∼30.6 g) (to not risk injuring the mouse skin) using an artificial mouse claw in a scratch position (Fig. S1, http://links.lww.com/PAIN/C154, see next paragraph for information regarding the artificial claw). The pressure applied was over the pain threshold of 260 mN.^43^ The stimulus was applied to the calf as we aimed to analyze the lumbar region of the spinal cord. For pinching, the mouse was pinched 5 times for 5 seconds using a tweezer with a 5-second resting period between each pinch episode. When 40 minutes had passed since the stimulus, the mouse was injected with 0.1 mL of a 1:1 mixture of Domitor (Domitor, 1 mg/mL, Orion Pharma) and Ketamine (Ketalar, 10 mg/mL, Pfizer) followed by perfusion and tissue preparation for RNAscope as described above.

### 2.13. Generation of an artificial mouse claw for scratching stimulation

The right hind paw isolated from the tibia and fibula was put in a “scratching” clawed position and was thereafter placed in 4% FA (Histolab) at 4°C for 4 days, whereafter the lower part of the fixated paw was mounted in 4% agarose (VWR, Stockholm, Sweden).

### 2.14. Microcomputed tomography

The fixated mounted paw was scanned using microcomputed tomography (μCT, SkyScan 1172, Bruker MicroCT, Kontich, Belgium). The paw was kept in saline solution until scanning. The mounted part of the paw was connected to the sample stage of the μCT, and the paw was scanned in air. The scanner operated at a voltage of 60 kV and a current of 167 μA. Images were acquired with an isotropic pixel size of 9.8 μm. The scan duration was 17 minutes. Reconstruction of cross-sections was done using the software package NRecon (Bruker MicroCT).

### 2.15. Image processing, computer-aided design, and 3D printing

The µCT data were processed in the 3DSlicer software (http://www.slicer.org/) to include all tissues in the paw and exported as an STL file. The handle for the scratching tool was drawn and fused to the paw STL using Fusion 360 (Autodesk Inc, San Francisco, CA) and exported as a single STL file. Slicing and generation of toolpaths for 3D printing were done using Preform (Formlabs, Somerville, MA) and printed on a Form 3 stereolithographic 3D printer (Formlabs) at 50-µm layers in White V4 resin (Formlabs). The printed part was processed according to the manufacturer's recommendations,^13,18,19,26,27,49,50^ resulting in the “Mouse Scratcher V1” (Fig. S1, http://links.lww.com/PAIN/C154). For coordinates to create a 3D-printed artificial mouse claw, the files “MouseScratcher_vi_formfile.form” and “MouseScratcher_v1.stl” are available upon request.

### 2.16. Hargreaves stimulation for fos detection

Three adult C57BL/6J mice (2 females and 1 male, 11-17 weeks old) were first acclimatized for 60 minutes in Hargreaves set up (transparent acrylic glass chambers on a glass floor) to minimize stress. The mice were stimulated 3 times with 5 minutes allowed to pass between each stimulation by directing the Hargreaves heat source (IITC Life Science, Los Angeles, CA) guided by a light pointer on the plantar surface of the right hind paw. The thermal source was on until the mouse withdrew/flinched its paw and the cut-off time was set to 20 seconds to minimize the risk of tissue damage. Forty minutes after completion of Hargreaves stimulation, the mouse was injected intraperitoneally with 0.7 to 0.8 mL ketamine (Ketalar, 10 mg/mL, Pfizer) and medetomidine (Domitor, 1 mg/mL, Orion Pharma) (1:1), followed by perfusion and tissue preparation for RNAscope as described below.

### 2.17. Sensory behavioral tests

All behavioral tests were performed on adult (>6 weeks) mice of both sexes. Experimental groups were matched in terms of age and sex, and when possible, littermate controls were used. Experiments were performed in a controlled environment at 20 to 24°C, 45%-65% humidity, and a 12-hour day/night cycle. The observers were blind to the viral vectors used. The same experimenter scored all observations within an experiment. The different experimental protocols are illustrated in Figure S2, http://links.lww.com/PAIN/C154.

### 2.18. Basal behavioral observation after chemogenetic activation or silencing of Urocortin 3-Cre neurons

Ucn3-Cre mice unilaterally injected in L4-L5 with AAV8.hsyn-DIO-hM3D(Gq)-mCherry (3 males and 3 females, 16-22 weeks) or AAV8.hsyn-DIO-mCherry (control; 4 females, 16-24 weeks) were injected intraperitoneally with 0.5 or 5 mg/kg clozapine-N-oxide (CNO, AK Scientific, Union City, CA, dissolved in 1% DMSO in sterile saline) and allowed approximately 10 minutes of acclimatization in the behavioral setup (transparent rectangular plastic compartment). The basal behavior of the mice following CNO administration was thereafter recorded for 30 minutes; after completion, the mice were returned to their home cage. In the Ucn3-Cre silencing basal recordings, mice injected with AAV8.hsyn-DIO-hM4D(Gi)-mCherry or AAV8.hsyn-DIO-mCherry (12 females and 8 males, 8-19 weeks old when injected) were acclimatized for 20 minutes in a plastic cylinder setup (diameter: 19 cm, height: 29 cm, surface area: 283 cm^2^) with a mirror to obtain 360° view before CNO injection (5 mg/kg). The basal behaviors were video recorded for 60 minutes, and the mice were returned to their respective home cage after completion of the recording. The duration and frequency of targeted behaviors were analyzed for total recording. The same experimenter scored all the behavior recordings manually using the software AniTracker v1.2. The licking/biting of the ipsilateral leg and paw were scored as 1 behavior, for which the episodes were scored when contact between face and skin area was clearly visualized.

### 2.19. Compound 48/80-induced behavior

Adult Ucn3-Cre mice (12 females and 8 males), injected with AAV8.hsyn-DIO-hM4D(Gi)-mCherry or AAV8.hsyn-DIO-mCherry at an age of 8 to 19 weeks, were administrated intraperitoneally with freshly prepared 5 mg/kg CNO (AK Scientific, dissolved in 1% DMSO in sterile saline) and returned to home cage for 30 minutes. The mice were thereafter acclimatized for 10 minutes in a plastic cylinder setup (see above), followed by subcutaneous injection of 10 μL Compound 48/80 (10 μg; Sigma-Aldrich, Cat# C2313, diluted in sterile saline) in the right dorsolateral calf. We chose to place the injections subcutaneously to increase reproducibility and reduce the risk of injection-induced injuries to the skin. The mice were returned to the setup and video recorded for 30 minutes. The experimenter was present in the room during the recording. The number of bouts and duration of biting/licking episodes of the injected area were scored manually using the software AniTracker v1.2.

### 2.20. Von Frey

The mice (7 females and 6 males, aged 6-14 weeks) were placed in transparent plastic chambers on an elevated metallic mesh floor for 90 minutes before the initiation of the experiment for acclimatization. Approximately 30 minutes before the first measurement, the mice were injected intraperitoneally with 5 mg/kg CNO (AK Scientific, dissolved in 1% DMSO in sterile saline). Von Frey filaments (Scientific Marketing Associates, London, United Kingdom) were applied on the plantar surface of the hind paw of each mouse according to the Chaplan up–down paradigm.^9^ A lift of the treated paw was considered a reaction to the applied filament and a thinner filament was applied subsequently, while a lack of reaction resulted in the application of a thicker filament. Every observation started with 0.6 g filament and the experiment was ended once 6 measurements were obtained around the 50% threshold. The Dixon method was used to calculate the 50% threshold for each mouse.

### 2.21. Hargreaves

The mice (7 females and 6 males, aged 8-16 weeks) were placed in transparent plastic chambers that were placed on top of a glass floor for 90 minutes until no exploratory behavior was observed. Approximately 30 minutes before the first measurement, the mice were injected intraperitoneally with 5 mg/kg CNO (AK Scientific, dissolved in 1% DMSO in sterile saline). A thermal laser beam (IITC Life Science) was directed towards their plantar hind paws and the time until paw withdrawal was noted. The test had a cut-off time of 20 seconds to avoid tissue damage and was repeated at least 3 times with 5 minutes between each observation. A mean withdrawal time was calculated for each mouse.

### 2.22. Experimental design and statistical analyses

All behavioral testing was performed a minimum of 11 days after viral injection to allow sufficient expression of viral vector genes. Ucn3-Cre.hM3Dq mice and their Ucn3-Cre.mCherry controls were included in 2 basal behavioral analyses/mice with 7 days between. Ucn3-Cre.hM4Di mice and their Ucn3-Cre.mCherry controls were included in a maximum of 2 behavioral tests (including basal recording) with a minimum of 2 days between the tests. The basal recording was conducted first, followed by the Compound 48/80 test. The von Frey test was conducted first followed by the Hargreaves test. The mice were returned to their home cages after each completed behavioral test. No mice were excluded from the behavioral analyses presented except mice where the viral vector/injection did not work or initial experiments without blinding and sex- and age-balanced groups. No randomization was used. Mice were arbitrarily assigned to different treatments (eg, injections of viral vectors) based on sex and age. All behavioral experiments were conducted by female experimenters, who were blinded to viral vectors (control vs chemogenetic). In the sensory stimulation tests to examine *fos*, the mice were arbitrarily assigned to the different stimuli, and we ensured that both sexes were used in the testing.

The number of mice per behavioral and in situ experiment was not based on any statistical calculations before the experiments. Sample sizes are in line with similar studies in the field.^7,15,24^

### 2.23. Statistics

All data were tested for normality using D'Agostino & Pearson normality test, Shapiro-Wilk normality test, or KS normality test before continuing with an appropriate statistical test. All statistics were performed using GraphPad Prism v9.3.1., except for the single-cell analysis that was performed using SCANPY 1.9.1.

#### 2.23.1. Immunohistochemistry of Urocortin 3-Cre rabies retrograde traced tissue

Images of transverse sections of traced spinal cords were aligned as stacks in ImageJ. The mediolateral and dorsoventral coordinates of traced cells were plotted with ImageJ's Cell counter, and the rostrocaudal distance between sections was calculated based on the thickness of sections. The mean coordinate for all starter cells was calculated for each mouse, and the position of each traced cell was compared with this coordinate in the mediolateral, dorsoventral, and rostrocaudal direction, as well as the absolute distance in 3 dimensions (3D). The data were checked for normality using the Shapiro-Wilk test. Differences in distance (mm) between the traced excitatory and inhibitory expressing cells were then calculated by 1-way ANOVA with Šídák's multiple comparisons test.

#### 2.23.2. Single-cell Urocortin 3 analysis of the Zeisel et al. (2018) dataset

The top differentially expressed genes between *Ucn3* expressing SCGLU7 and SCGLU9 clusters were calculated and ranked using unpaired *t* test. The *P*-values were further adjusted with the Benjamini-Hochberg procedure to decrease the false discovery rate (FDR).

#### 2.23.3. Electrophysiology

Paired 2-tailed *t* test was used for tonic current comparison.

#### 2.23.4. RNAscope

Number differences between the contralateral and ipsilateral (stimuli-evoked) *fos(+)* or the *Ucn3(+)fos(+)* cells for each stimuli were calculated using paired 2-tailed *t* test. Eight of the 17 sections used to analyze the pinch stimuli were older than 3 months. However, when comparing the contralateral and ipsilateral sides, a paired 2-tailed *t* test was used, thereby compensating for the possible impact mRNA degradation may have on the result. Compound 48/80 was also compared with saline (control) by 1-way ANOVA with Bonferroni multiple comparisons.

#### 2.23.5. Behavioral tests

Chemogenetics: Basal activation: One-way ANOVA with post hoc Šidák's multiple comparisons test, basal inhibition: Kruskal-Wallis with Dunn's multiple comparisons test, Compound 48/80-induced behavior, duration and frequency: Duration Mann-Whitney *U* test and frequency Unpaired two-tailed t test, Hargreaves: Mann-Whitney *U* test. *Von Frey*: Unpaired two-tailed t-test. All data is plotted as mean ± SEM. * *P* < 0.05.

## 3. Results

### 3.1. Spinal lumbar Urocortin 3-Cre neurons express Urocortin 3 and are excitatory

**Figure 1.**
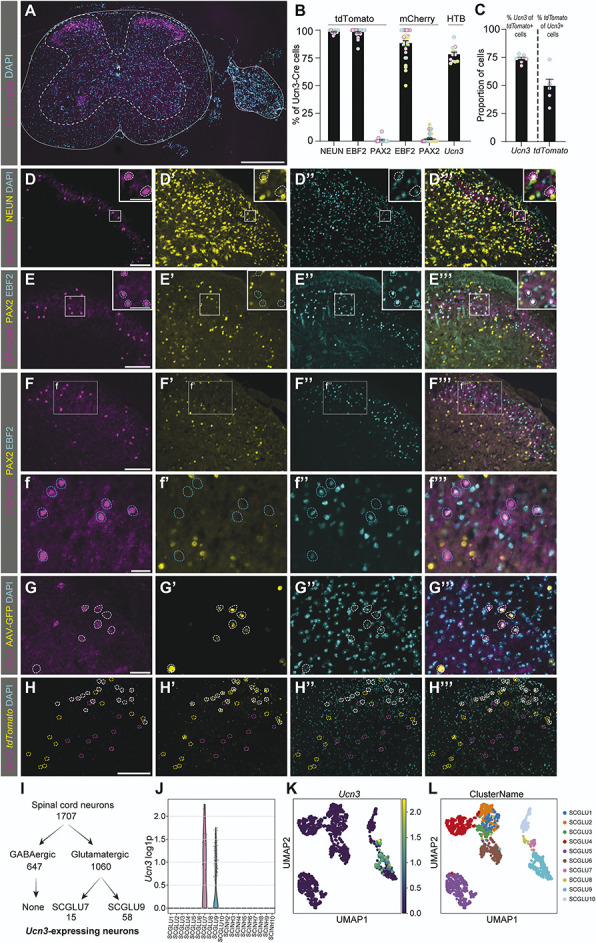
Ucn3-Cre is expressed in spinal excitatory neurons in the dorsal horn. Analysis of Ucn3-Cre expression using Ucn3-Cre;tdTomato reporter mice or using reporter virus. (A) Ucn3-Cre expression in the lumbar spinal cord and dorsal root ganglion is confined to the dorsal horn of the spinal cord. (B) Bar chart displaying the percentage of Ucn3-Cre-expressing cells overlapping with the respective marker. Each dot represents 1 section from a specific mouse, where different mice are coded with different colors (plotted as mean ± SEM, where the mean is based on the number of sections). (C) Bar chart displaying the percentage of Ucn3-Cre;tdTomato cells overlapping with *Ucn3* or *tdTomato*, where the different mice are coded with different colors (plotted as mean ± SEM, where the mean is based on the number of sections). (D) Spinal Ucn3-Cre cells express the neuronal marker NEUN (591/599 cells, n sections = 10, n = 2 mice). (E) Ucn3-Cre cells express the excitatory marker EBF2 (553/564 cells, n sections = 24, n = 2 mice) but rarely the inhibitory marker PAX2 (3/287 cells, n sections = 12, n = 2 mice). (F) Ucn3-Cre cells marked by the AAV8/hSyn-DIO-mCherry virus express the excitatory marker EBF2 (379/429 cells, n sections = 29, n = 3 mice) but barely the inhibitory marker PAX2 (11/429 cells, n sections = 29, n = 3 mice). (G) Spinal Ucn3-Cre cells express *Ucn3* mRNA (469/612 of virally labeled Ucn3-Cre.HTB cells, n sections = 12, n = 3 mice). (H) Spinal Ucn3-Cre;tdTomato cells, in Ucn3-Cre;tdTomato reporter mice, express *Ucn3* mRNA (258/351 cells, n sections = 6, n = 2 mice) and *tdTomato* mRNA (258/549 cells, n sections = 6, n = 2 mice); however, single-labeled cells are present. White dashed lines denote triple-labeled cells, cyan dashed lines indicate Ucn3-Cre;tdTomato cells that only overlap with the marker in the cyan channel, and magenta dashed lines indicate cells that only express the marker in the magenta channel, whereas yellow dashed lines indicate cells that only express the marker in the yellow channel. (I-L) Single-cell mRNA analysis of *Ucn3*-expressing cells in the Zeisel et al. dataset.^68^ (I) *Ucn3* was detected in the glutamatergic SCGLU7 and SCGLU9 clusters, but not in GABAergic neurons (*Ucn3* was considered expressed if log1 *P* > 0.5). (J) Violin plot of *Ucn3* showing restricted expression in excitatory SCGLU7 and SCGLU9 spinal cord neurons. (K-L) UMAP topography plot of Zeisel et al. (2018) depicts excitatory neurons showing an expressional similarity between *Ucn3*-expressing cells (K) and SCGLU7 and SCGLU9 neurons (L) as indicated by their close proximity. Scale bars: (A) 500 µm, (D, F, and H) 200 µm with enlargements 67 µm, (E) 200 µm with enlargements 100 µm, and (G) 50 µm. UCN3, Urocortin 3; UMAP, uniform manifold approximation and projection.

To investigate the identity of UCN3 cells, the Ucn3-Cre line was crossed with the reporter line tdTomato or injected with reporter virus (Fig. 1A-H). In the spinal cord, Cre-dependent tdTomato expression was identified in a band of cells in the dorsal horn (Fig. 1A) that overlapped with the neuronal marker neuronal nuclei (NEUN) (99 ± 0.5%) (Fig. 1B, D). Further analyses in Ucn3-Cre;tdTomato mice using the excitatory marker EBF transcription factor 2 (EBF2)^24^ and the inhibitory marker paired box gene 2 (PAX2)^32^ identified 98 ± 0.8% of the Ucn3-Cre neurons as EBF2(+) and 0.96 ± 0.7% as PAX2(+) (Fig. 1B, E). To visualize only the neurons expressing Cre in the adult context, we injected adeno-associated virus (AAV)8/hSyn-DIO-mCherry reporter virus locally in the dorsal horn in adult Ucn3-Cre mice. The expression pattern of mCherry was similar to Ucn3-Cre;tdTomato neurons, as Ucn3-Cre;mCherry neurons highly coexpressed EBF2 (88 ± 2%) and overlapped less with PAX2 (2.2 ± 0.8%) (Fig. 1B, F). An earlier study did not detect *Ucn3* in the spinal cord,^45^ and hence we wanted to address whether spinal Ucn3-Cre expression was due to continued activity of the *Ucn3* promotor or an aberrant expression of Cre. Ucn3-Cre activity was visualized with an AAVDJ/Ef1a-DIO-HTB virus, and expression of *Ucn3* was detected in 76.1 ± 1.9% of the Ucn3-Cre;HTB neurons (Fig. 1B, G). Moreover, the tdTomato reporter line was used to visualize the anatomical location of the *Ucn3* mRNA. Collectively, 71.3 ± 1.59% of the *Ucn3*(+) cells expressed *tdTomato,* and 49.7 ± 4.3% of the *tdTomato*(+) cells were co-labeled with *Ucn3* (Fig. 1C, H). Control sections tdTomato(+)Ucn3-Cre(−) showed 2.2 ± 1.3% co-labeled cells (data not shown). To challenge the finding that lumbar spinal *Ucn3* neurons are excitatory, a single-cell analysis based on the Zeisel et al. spinal cord dataset^68^ was performed. The analysis identified *Ucn3* in 2 subtypes of glutamatergic neurons in the dorsal spinal cord, SCGLUT7 and SCGLUT9 (Fig. 1I-L). This analysis collectively indicates that the adult spinal lumbar Ucn3-Cre population expresses *Ucn3* to a high extent and is predominately excitatory.

### 3.2. Spinal lumbar Urocortin 3 neurons receive input from local inhibitory and excitatory neurons and are under tonic inhibition

**Figure 2.**
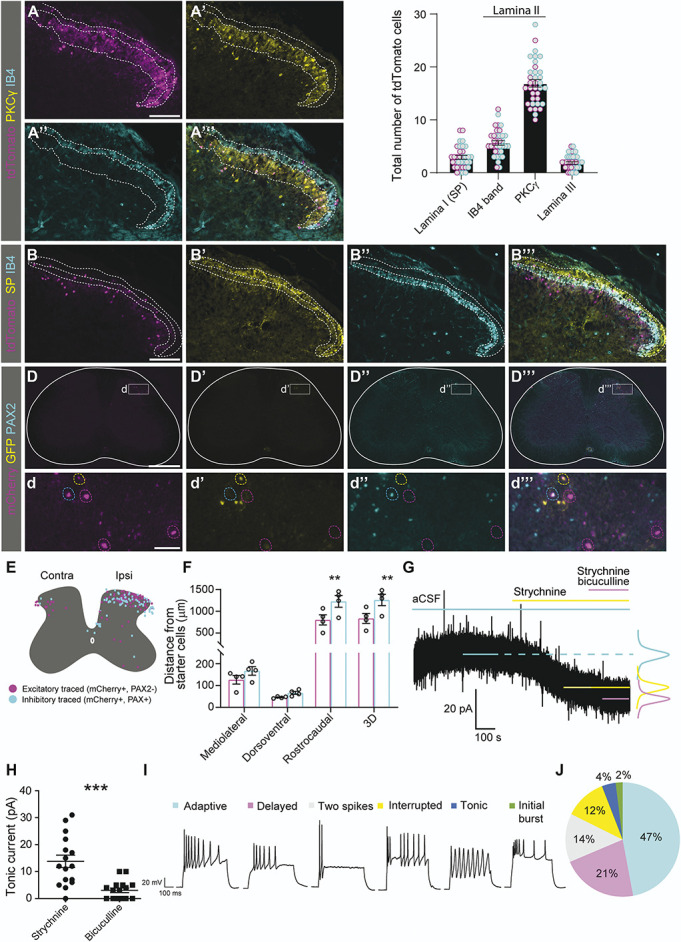
Lumbar Ucn3-Cre neurons have neurites in the dorsal horn, are contacted by local excitatory and inhibitory neurons, and are under tonic inhibition. The area of Ucn3-Cre-expressing cell neurites was assessed through the intensity of red background in Ucn3-Cre;tdTomato reporter mice. (A) The dotted line encloses the area of highest tdTomato intensity, representing the location of most neurites. This area co-localizes with the bands of PKCγ and IB4 staining in lamina II (A′-A‴). (B-C) Cell bodies of Ucn3-Cre cells are found mostly within the PKCγ band of inner lamina II (n = 45 sections, n = 2 mice), with fewer cell bodies found deeper in the dorsal horn (n = 35 sections, n = 2 mice) or in the IB4 band of lamina II_i_ (n = 35 sections, n = 2 mice) or in lamina I (as identified by substance P (SP) immunoreactivity, n = 35 sections, n = 2 mice). The dotted lines represent borders of PKCγ, IB4, and SP immunoreactivity (plotted as mean ± SEM, where the mean is based on the number of sections). (D-E) Retrograde rabies viral tracing found GFP(+)mCherry(+) starter cells in the dorsal horn in the area of Ucn3-Cre cells and did not express PAX2 (the location of all individual starter cells can be found in Fig. S3, http://links.lww.com/PAIN/C154). Trans-synaptic mCherry retrogradely traced cells in the spinal cord were found primarily in the ipsi- and contralateral dorsal horn. (F) The traced inhibitory and excitatory cells differed in their distance relative to the starter cells in the mediolateral and rostrocaudal axis, with excitatory cells located closer to the starter cells than inhibitory cells in the rostrocaudal axis ((plotted as mean ± SEM, where the mean is based on the number of mice), n sections = 357, n = 4 mice). One-way ANOVA with Šidák multiple comparisons test. (G) The electrophysiological properties of lumbar Ucn3-Cre cells were studied in Ucn3-Cre;tdTomato reporter mice. The application of strychnine (glycine receptor antagonist) and bicuculline (GABA receptor antagonist) revealed glycinergic and GABAergic inhibitory tonic currents, respectively, as the result of depolarization (downshifting of the baseline). (H) Measured inhibitory tonic currents. Circular dots correspond to glycinergic inhibitory tonic currents, while squares are the GABAergic tonic currents (mean ± SEM, where the mean is based on the number of cells, 2-tailed *t*-tests, ** *P* < 0.01, *** *P* < 0.001). (I-J) AP firing patterns of Ucn3-Cre neurons (n = 51 neurons from 29 mice). The pie chart displays the distribution of AP firing patterns, and representative AP firing patterns are shown on the right. Scale bars: A 200 µm, applies to (A-B). (D) 500 µm, d 50 µm. AP, action potential; GFP, green fluorescent protein; PKCγ, protein kinase C gamma; UCN3, Urocortin 3.

The function of cells in the dorsal horn is highly related to their laminar distribution.^1,24^ Therefore, we characterized the location of the cell bodies and projections of spinal lumbar Ucn3-Cre neurons within the dorsal horn (Fig. 2A-C) of Ucn3-Cre; tdTomato reporter mice. Most of the somata and neurites of Ucn3-Cre;tdTomato neurons were found to overlap with lamina II_i_ projections of PKCγ neurons and IB4(+) primary afferents (Fig. 2A, C). A few Ucn3-Cre somata were found ventral of the PKCγ band (Fig. 2A, C), and within lamina I and II_outer_ (II_o_), identified by substance P (SP) primary afferent projections (Fig. 2B-C), indicating that lumbar Ucn3-Cre neurons are mainly localized within lamina II_i_.

To study the synaptic input to Ucn3-Cre neurons from spinal interneurons, descending neurons from the brain, and primary afferents, we performed rabies virus-based retrograde trans-synaptic tracing (Fig. 2D-E), where Cre(−) littermates (n = 2), injected using the same protocol, were used as controls (Fig. S3A, http://links.lww.com/PAIN/C154). We found Cre-dependent expression of GFP in the dorsal horn of injected Ucn3-Cre mice in a similar pattern as observed in Figure 2A-B, including mCherry(+)GFP(+) cells (presumed starter cells) (Figure 2D, Figure S3B-C, http://links.lww.com/PAIN/C154). Green fluorescent protein was not detected in control mice (analysis of every sixth section, Fig. S3A, http://links.lww.com/PAIN/C154). The highest density of cells expressing mCherry in the Ucn3-Cre mice was found in the ipsilateral dorsal horn, where 78 ± 7 cells per mouse were found upon analysis of every sixth section. Moreover, few cells were found in the ipsilateral ventral horn and in the contralateral dorsal horn (Fig. 2E). Traced neurons were almost exclusively found within the lumbar enlargement of the spinal cord, with only 2 cells in the thoracic segments, while none were found in the cervical nor the sacral regions.

PAX2(+) traced cells were found in both the ipsilateral and the contralateral dorsal horn of Ucn3-Cre mice (Fig. 2E). Further analysis of the ipsilateral dorsal horn showed that PAX2(+) traced cells resided farther away from the starter cells than PAX2(−) traced cells (*P* = 0.0091 (3D), *P* = 0.0095 (rostrocaudal), Figure 2F, Figure S3D, http://links.lww.com/PAIN/C154). In controls, when analyzing every sixth section, 7 mCherry(+)GFP(−) cells were found in the ipsilateral dorsal and 1 cell in the ipsilateral ventral horn (in total 8 cells, 4 per mouse). When examining every second section of the brains (70 µm thick) from the Ucn3-Cre(+) and the control mice, no mCherry fluorescent signal was detected.

To understand the influence of pre-synaptic inhibitory neurons on lumbar Ucn3-Cre neurons, whole-cell patch-clamp recordings were performed on Ucn3-Cre;tdTomato neurons in Ucn3-Cre;tdTomato reporter mice (Fig. 2G-J). In addition to the intrinsic properties, tonic inhibitory input plays an important regulatory role in the excitability of a neuron,^34^ and hence, patched neurons, at −60 mV in voltage-clamp mode, were perfused with strychnine (glycine receptor antagonist) and bicuculline (GABA receptor antagonist). Ucn3-Cre neurons were found to receive both glycine receptor- and GABA receptor-mediated tonic inhibition (Fig. 2G), where the glycine receptor-mediated tonic inhibition current was higher than the GABA (Fig. 2H, *P* = 0.0005). Tonic inhibition has also been observed in other somatosensory neurons.^16^

In addition, Ucn3-Cre neurons demonstrated varieties of action potential (AP) firing patterns, among which adaptive AP patterns comprised almost half (47%) of the patched neurons in Ucn3-Cre;tdTomato reporter mice (Fig. 2I-J, n = 51). A similar distribution of Ucn3-Cre neuron AP patterns has previously been shown,^33^ although the term used there for adaptive APs was “initial bursting.” The recorded neurons showed an average resting membrane potential at −58.7 ± 1.3 mV, rheobase at 33.6 ± 4.3 pA, and membrane resistance at 717 ± 58.2 MΩ. Conclusively, lumbar spinal Ucn3-Cre neurons receive input from local spinal excitatory and inhibitory interneurons and are under tonic inhibition.

### 3.3. Lumbar Urocortin 3-Cre neurons receive input from primarily Aβ and C fibers

**Figure 3.**
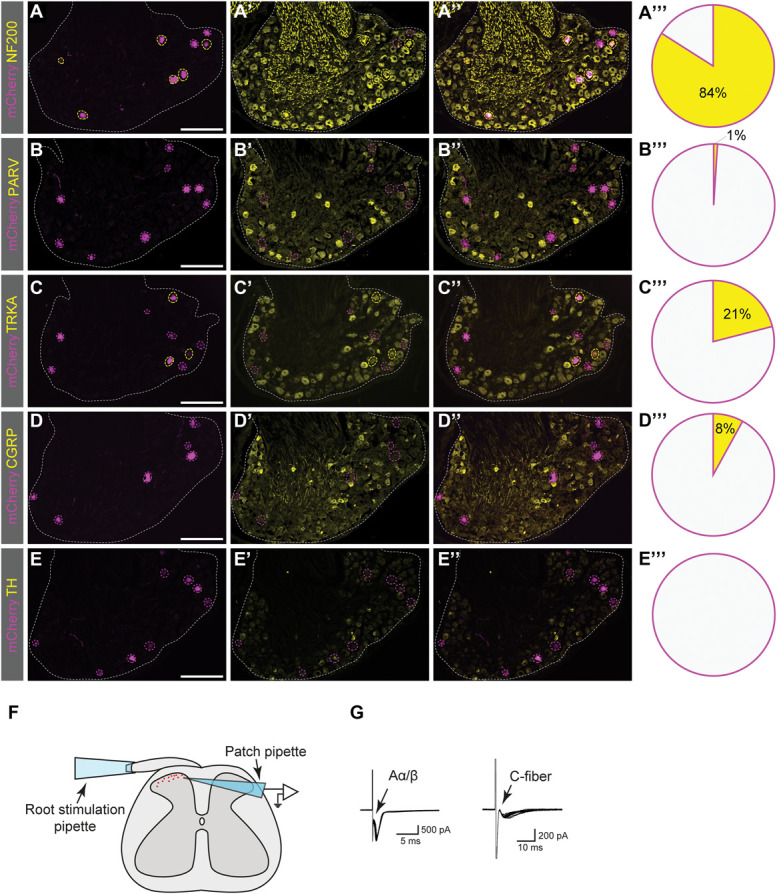
Retrograde trans-synaptic viral tracing and electrophysiological recordings of spinal lumbar Ucn3-Cre neurons show input from primarily Aβ and C fibers. (A) A majority (84%) of traced cells in the DRG expressed NF200, a marker for myelinated primary afferents (61/73 cells, n = 27 sections, n = 3 mice). (B) PARV, a marker for proprioceptive cells, was expressed in only 1% of traced DRG cells (1/102 cells, n = 35 sections, n = 3 mice). (C) TRKA, a marker for both myelinated Aδ and unmyelinated C fibers, was expressed in 21% of traced cells (20/97 cells, n = 36 sections, n = 4 mice). (D) CGRP, a marker for unmyelinated, mainly nociceptive fibers, was expressed in 8% of traced DRG cells (9/112 cells, n = 34 sections, n = 4 mice). (E) TH, a marker for unmyelinated cells mediating pleasant touch, was not found in any traced DRG cells (0%, 0/84 cells, n = 28 sections, n = 4 mice). (F) A schematic illustration of patch-clamp recording combined with root stimulation (figure to the left). (G) Representative traces of monosynaptic connection from Aα/Aβ fiber (left) and C fiber (right) to a patched Ucn3-Cre neurons (n = 22 neurons, each trace comprises 20 superimposed recordings) in Ucn3-Cre;tdTomato reporter mice. Scale bar: 200 µm (A-E). CGRP, calcitonin gene-related peptide; DRG, dorsal root ganglia; PARV, parvalbumin; TH, tyrosine hydroxylase; TRKA, tropomyosin receptor kinase A; UCN3, Urocortin 3.

The retrograde trans-synaptic viral tracing analysis identified traced neurons in the L4 and L5 DRG, and in smaller numbers in the L3 and L6 DRG. In controls, no mCherry expression was detected in the DRG (n = 2 mice, 20 sections). Costaining with sensory neuron population markers revealed that most of the traced DRG neurons in Ucn3-Cre(+) mice belonged to the myelinated subset expressing Neurofilament 200 (NF200) (84%) (Fig. 3A). However, only 1% of traced DRG neurons were identified as proprioceptive NF200(+) neurons by coexpression of PARV (Fig. 3B). This indicates that the traced myelinated neurons primarily do not belong to the Aα proprioceptive subpopulation. Among the lumbar spinal Ucn3-Cre traced neurons, 21% expressed tropomyosin receptor kinase A (TRKA) (Fig. 3C), which marks a subset of small unmyelinated mainly nociceptive neurons as well as a subset of large myelinated NF200(+) neurons with Aδ fibers.^5^ Our traced TRKA(+) cells were larger than other TRKA(+) cells (1549 vs 1259 µm^2^, *P* = 0.049, Fig. S3E, http://links.lww.com/PAIN/C154). Moreover, 8% of traced cells coexpressed Calcitonin gene-related peptide (CGRP) (Fig. 3D), a marker of a population of unmyelinated peptidergic neurons (C fibers), largely coexpressing TRKA in small NF200(−) neurons.^4^ No traced cells expressed tyrosine hydroxylase (TH), a marker expressed in low-threshold NF200(−) mechanosensitive DRG neurons^37^ (Fig. 3E). Taken together, these results indicate that spinal lumbar Ucn3-Cre neurons receive robust peripheral input from mainly nonproprioceptive sensory NF200(+)-DRG neurons.

To further investigate which type of fibers signal to Ucn3-Cre neurons, the dorsal root was activated using a root stimulation pipette, while patch-clamp recording was performed on individual lumbar spinal Ucn3-Cre;tdTomato neurons in Ucn3-Cre; tdTomato reporter mice (Fig. 3F). More than half of the patched neurons (13 out of 22) received monosynaptic inputs from either Aα/Aβ fibers (1 neuron) or C fibers (1 neuron), or both Aα/Aβ and C fibers (11 neurons) (Fig. 3G). The remaining neurons (9 out of 22) did not demonstrate any monosynaptic connection to afferent fibers. It should be noted that the preparation of spinal cord slices can, in fact, disrupt the connection; therefore, the missing of monosynaptic input from primary afferents could be due to either the lack of connection or damage during the preparation. In summary, the Ucn3-Cre population receives monosynaptic connections from primarily Aβ and C fibers.

### 3.4. Spinal lumbar Urocortin 3 neurons are activated by Compound 48/80 or scratching

To investigate the sensory role of spinal lumbar *Ucn3* neurons, we performed sensory stimulations in anesthetized or awake C57BL/6J mice followed by RNAscope analysis^63^ of *fos*^56^ and *Ucn3* in the L5/L6 dorsal spinal cord. Mice were subjected to either subcutaneous injection of saline (control) or Compound 48/80 in the right dorsolateral calf, noxious mechanical (stimulation pinch or scratching (Fig. S1, http://links.lww.com/PAIN/C154)) of the right dorsolateral calf, or thermal (Hargreaves) stimulation of the right hind paw. To prevent transcriptional influences from noci- and/or prurifensive behaviors, all stimulations were performed under urethane anesthesia except for Hargreaves, which was performed on awake freely moving mice.

**Figure 4.**
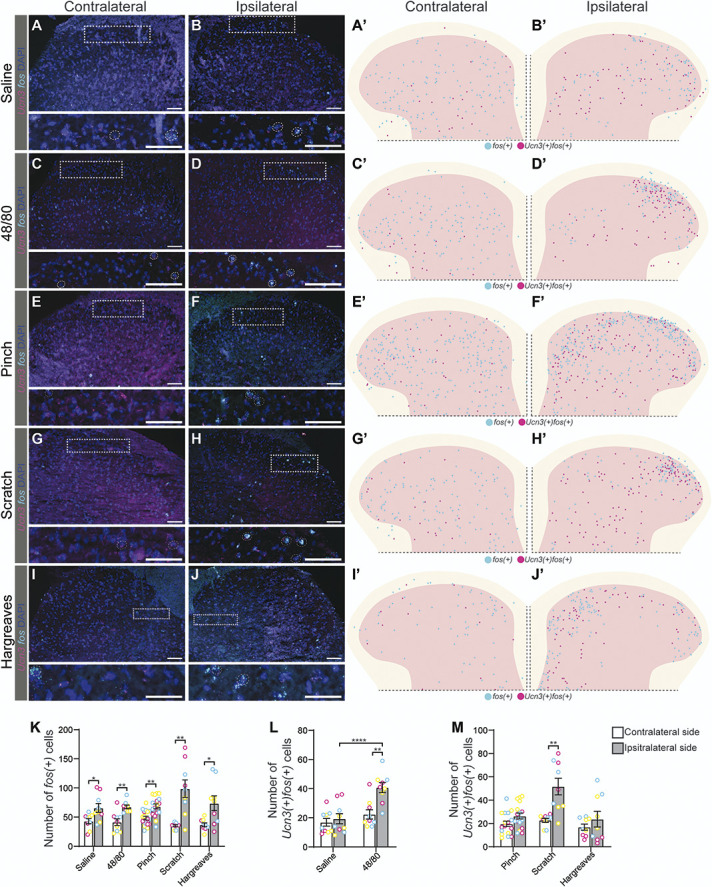
A subpopulation of spinal lumbar *Ucn3* neurons expresses *fos* after Compound 48/80 or scratching. Mice (n = 3 of mixed sex/test) were subjected to different sensory stimuli. (A-B′) Saline, (C-D′) Compound 48/80, (E-F′) pinch, (G-H′) scratch, or (I-J′) Hargreaves, to investigate the coexpression of *Ucn3* (magenta) and *fos* (cyan) (DAPI (dark blue)). For separate channels, see Figure S4-5, http://links.lww.com/PAIN/C154. (A-J) Representative images of the contralateral and ipsilateral (stimulated side) dorsal horns for each stimulus, with close-ups. Examples of coexpressing cells are indicated by a dashed white circle. Scale bars: 100 µm. (A′-J′) Schematic illustrations of the cells expressing *Ucn3* and *fos,* each cell is illustrated by a dot. *Fos(+)* cells (cyan) and *Ucn3(+)fos(+)* cells (magenta) (contralateral n = 9, except for saline (n = 7) and pinch (n = 17), ipsilateral n = 9 for all stimuli, except pinch (n = 17)). (K-M) The average number of *fos*(+) cells per dorsal horn (mean ± SEM, where the mean is based on the number of sections) was calculated for each stimulus on the contralateral (white bar) and ipsilateral side (grey bar). Individual mice are marked with magenta, yellow, and cyan in (K-M) to display the spread between sections and mice. (K) Saline, contralateral: 43 ± 4 (387) and ipsilateral: 66 ± 8 (592); Compound 48/80, contralateral: 41 ± 6 (373) and ipsilateral: 67 ± 3 (607); pinch, contralateral: 45 ± 4 (768) and ipsilateral: 63 ± 4 (1063); artificial scratching, contralateral: 35 ± 2 (345) and ipsilateral: 99 ± 15 (889); and noxious heat stimulation (Hargreaves), contralateral: 37 ± 3 (332) and ipsilateral: 74 ± 13 (664) *fos(+)* cells. When comparing the contralateral to the ipsilateral side using paired two-tailed *t* test, saline (*P* = 0.035), Compound 48/80 (*P* = 0.0034), pinch (*P* = 0.0063), scratch (*P* = 0.0021), and Hargreaves (*P* = 0.023) had a greater number of *fos(+)* cells on the ipsilateral (stimulated) side. (L-M) The average number of *Ucn3(+)fos(+)* cells for each stimulus was counted on the contralateral and ipsilateral sides. Saline, contralateral: 17 ± 3 (152) and ipsilateral: 19 ± 4 (174); Compound 48/80, contralateral: 22 ± 3 (201) and ipsilateral: 41 ± 3 (366); pinch, contralateral: 19 ± 3 (320) and ipsilateral: 26 ± 3 (432); artificial scratching, contralateral: 28 ± 2 (204) and ipsilateral: 52 ± 7 (465); and noxious heat stimulation (Hargreaves), contralateral: 17 ± 3 (152) and ipsilateral: 24 ± 7 (213) *Ucn3(+)fos(+)* cells. (L) Compound 48/80 was found to have a greater number of cells on the ipsilateral side than the contralateral side (paired 2-tailed *t* test, *P* = 0.0018), as well as compared with the saline injection (control) (1-way ANOVA with Bonferroni multiple corrections, ipsilateral side of saline vs the ipsilateral side of Compound 48/80 *P* < 0.0001). (M) The average number of *Ucn3(+)fos(+)* cells for the mechanical stimuli and the Hargreaves test was calculated for the contralateral and ipsilateral sides. Artificial scratching was the only stimulus that had a higher *fos(+)* cell count on the ipsilateral side compared with the contralateral side (paired two-tailed *t* test, *P* = 0.0016). *P < 0.05, **P < 0.01, ****P < 0.0001. UCN3, Urocortin 3.

*Ucn3(+)* neurons were found widespread in the dorsal horn, but more densely expressed in the upper laminae of the contra- and ipsilateral dorsal horn, while *fos* displayed distinct stimuli-dependent patterns (Fig. 4A-J, Figs. S4-5, http://links.lww.com/PAIN/C154). More *fos(+)* cells were observed in the ipsilateral dorsal horn compared with the contralateral dorsal horn for saline (*P* = 0.035), Compound 48/80 (*P* = 0.0034), pinch (*P* = 0.0063), scratch (*P* = 0.0021), and Hargreaves (*P* = 0.023), (Fig. 4K). *Ucn3(+)fos(+)* cells were found to be greater in number in the ipsilateral dorsal horn than the contralateral dorsal horn for both Compound 48/80 (*P* = 0.0018, Fig. 4L) and artificial scratching (*P* = 0.0016, Fig. 4M). Furthermore, the average number of *Ucn3(+)fos(+)* cells in the ipsilateral dorsal horn after injection with Compound 48/80 was higher than the average number of *Ucn3(+)fos(+)* cells after saline injection (*P* < 0.0001, Fig. 4L). Taken together, these results indicate that spinal *Ucn3(+)* cells are activated by Compound 48/80 as well as scratching.

### 3.5. Spinal Urocortin 3 neurons are divided into 2, nonoverlapping, protein kinase C gamma- and calretinin/calbindin 2-expressing populations

**Figure 5.**
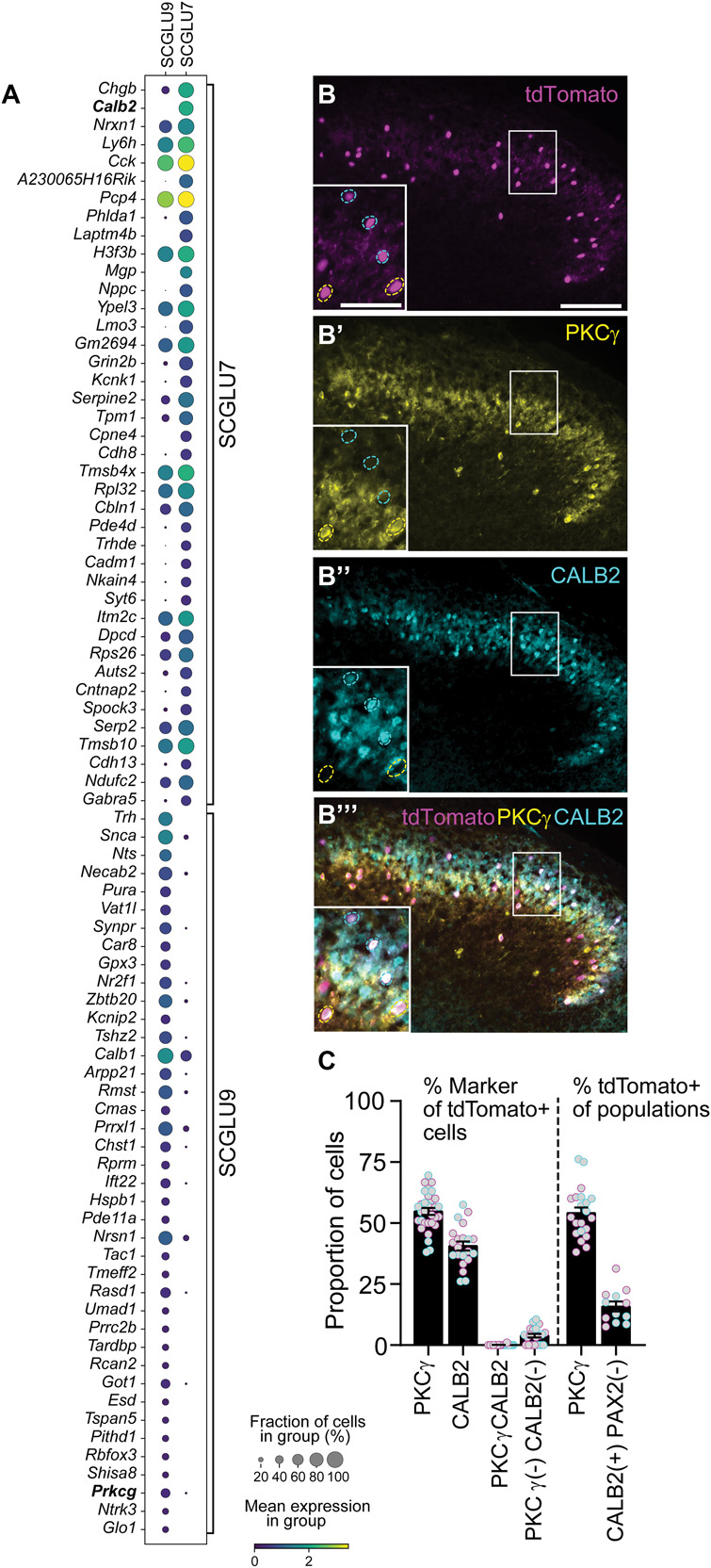
*Ucn3*-expressing cells represent 2 subpopulations expressing either *Prkcg* or *Calb2*. (A) Dot plot of the top differentially expressed genes in *Ucn3*-expressing SCGLU7 vs *Ucn3*-expressing SCGLU9 neurons. Among the top differently expressed genes in SCGLU7, *Calb2* is found, whereas *Prkcg* is a top differently expressed gene in SCGLU9 (*t* test, *P*-values further adjusted with the Benjamini-Hochberg procedure, FDR < 0.05). Ucn3-Cre cells can be divided into 2 subsets based on their coexpression of either PKCγ or CALB2 (Calbindin 2, also known as calretinin) immunoreactivity. (B-C) Immunohistochemical analysis of PKCγ (276/496 cells, n sections = 21, n = 2 mice) and CALB2 (203/496 cells, n sections = 21, n = 2 mice) reactivity in the Ucn3-Cre;tdTomato population, identified in Ucn3-Cre;tdTomato reporter mice, confirms the 2 nonoverlapping subpopulations, with practically no overlap (1/496 cells, n sections = 21, n = 2 mice). Ucn3-Cre activity was detected in approximately half (276/516 cells, n sections = 21, n = 2 mice) of the PKCγ cells and in 16% CALB2(+)PAX2(−) cells (80/496, n sections = 12, n = 2 mice) (Fig. S6, http://links.lww.com/PAIN/C154). Data are plotted as mean ± SEM, where the mean is based on the number of sections. Scale bar 200 µm in B, 100 µm for enlargements. FDR, false discovery rate; PKCγ, protein kinase C gamma; UCN3, Urocortin 3.

The *fos* analysis associated a subpopulation of the lumbar *Ucn3* neurons with the mechanical stimulus scratching. To further understand the molecular characteristics of spinal *Ucn3* neurons, we continued the analysis of the single-cell Zeisel et al. spinal cord dataset.^68^ *Ucn3* could be identified in 2 subtypes of glutamatergic neurons in the dorsal spinal cord: SCGLUT7 and SCGLUT9 (Fig. 1G-H). These populations displayed a high degree of expressional similarity as indicated by the close topographic proximity (Fig. 1I-J), but expressional differences between *Ucn3*-expressing SCGLUT7 and SCGLU9 were found (Fig. 5A). Among these differently expressed genes, we noticed the markers *Calb2*^21^ in SCGLUT7 and *Prkcg* (PKCγ)^52^ in SCGLUT9 (Fig. 5A), markers previously associated with the transmission of mechanical sensations.^38,47,48^ The complete list of top differentially expressed genes can be found in Table S1, http://links.lww.com/PAIN/C154. A closer analysis of *Prkcg* and *Calb2* expression on a single-cell level in the dataset confirmed that these markers are generally expressed in different cells, but *Calb2* expression was detected in some cells with a low level of *Prkcg* expression (Fig. S6A, http://links.lww.com/PAIN/C154). Immunostaining of sections from Ucn3-Cre;tdTomato reporter mice confirmed that most of the Ucn3-Cre(+) neurons expressed either PKCγ (56 ± 2%) or CALB2 (41 ± 2%), with practically no overlap (Fig. 5B-C). Ucn3-Cre-dependent tdTomato expression was detected in approximately half (53 ± 2%) of the PKCγ neurons and in 16 ± 2% of the excitatory CALB2(+)(PAX2(−) neurons (Figure 5B-C, Fig. S6B, http://links.lww.com/PAIN/C154). In conclusion, lumbar spinal Ucn3-Cre neurons can be divided into 2 practically nonoverlapping PKCγ- and CALB2-expressing populations.

### 3.6. Acute activation of spinal lumbar Urocortin 3-Cre neurons causes a biting/licking behavior, and inhibition attenuates Compound 48/80-induced behavior

**Figure 6.**
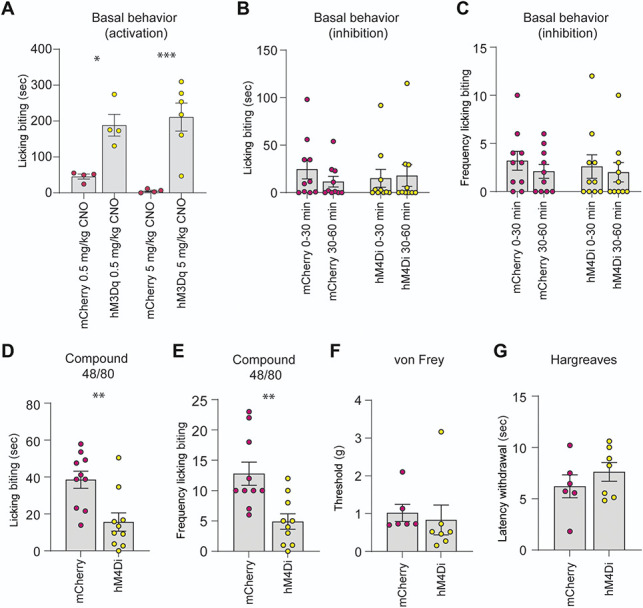
Chemogenetic activation of spinal lumbar Ucn3-Cre neurons causes a targeted behavior towards the corresponding dermatome, and inhibition alleviates Compound 48/80-evoked behavior. (A) Activating Ucn3-Cre neurons targeted with AAV8/hSyn-DIO-hM3D(Gq)-mCherry virus with CNO caused an increase in biting/licking (1-way ANOVA, post hoc Šidák multiple comparisons test, *P* = 0.0272, 0.5 mg/kg CNO; *P* = 0.0008, 5 mg/kg CNO) of the ipsilateral paw (corresponding dermatome). No significant behavioral difference was seen on the contralateral side, nor when the control virus without the hM3Dq receptor construct was used (Fig. S7, http://links.lww.com/PAIN/C154). (B-C) Silencing Ucn3-Cre neurons infected with AAV8/hSyn-DIO-hM4D(Gi)-mCherry virus with CNO did not affect the licking/biting duration (B) nor frequency (C) of the ipsilateral paw in the 0 to 30-minute and 30 to 60-minute intervals (Kruskal-Wallis test, post hoc Dunn multiple comparisons test, *P* > 0.99, 5 mg/kg CNO). (D-E) Silencing of Ucn3-Cre neurons attenuated the 48/80-evoked licking/biting response in terms of duration (D, Mann-Whitney *U* test, *P* = 0.0036, 5 mg/kg CNO) and frequency (E, unpaired *t* test, *P* = 0.0029, 5 mg/kg CNO). (F) Silencing Ucn3-Cre neurons did not cause alteration in mechanical threshold (Mann-Whitney *U* test, *P* = 0.1002, 5 mg/kg CNO). (G) The withdrawal response to noxious heat was not affected by the silencing of Ucn3-Cre neurons (unpaired *t* test, *P* = 0.3488, 5 mg/kg CNO). All data are plotted as mean ± SEM, where the mean is based on the number of mice. *P < 0.05. CNO, clozapine-N-oxide; UCN3, Urocortin 3.

To address the function of spinal lumbar Ucn3-Cre neurons in vivo, we used a chemogenetic approach and injected AAV8/hSyn-DIO-hM3D(Gq)-mCherry virus unilaterally in the lumbar spinal cord of Ucn3-Cre mice. Activating the hM3Dq receptor with CNO increased biting/licking of the ipsilateral leg and paw compared with Ucn3-Cre mice injected with the control virus lacking the hM3Dq receptor (AAV8/hSyn-DIO-mCherry; *P* = 0.0272, 0.5 mg/kg CNO; *P* = 0.0008, 5 mg/kg CNO; Fig. 6A), indicative of a perceived sensation.

To deduce the sensory function of spinal lumbar Ucn3-Cre neurons, mice injected with AAV8/hSyn-DIO-hM4D(Gi)-mCherry virus were subjected to different sensory stimuli while silencing Ucn3-Cre neurons. The results were compared with Ucn3-Cre mice injected with a control virus lacking the hM4Di receptor. First, to determine if chemogenetic silencing of Ucn3-Cre neurons evoked any sensory behaviors, the basal behavior phenotype was examined after CNO administration. Activation of the hM4Di receptor did not affect the targeted behavior towards the corresponding dermatome (licking/biting of the ipsilateral leg and paw), in the 0 to 30-minute and 30 to 60-minute intervals post CNO injection (0-30 minutes: duration *P* > 0.99, frequency (number of episodes) *P* > 0.99; 30-60 minutes: duration *P* > 0.99, frequency *P* > 0.99) compared with control AAV injected mice (Fig. 6B-C). To investigate the role of Ucn3-Cre spinal lumbar neurons in itch, the mice were injected with Compound 48/80 in the dorsolateral calf. Compound 48/80 degranulates mast cells and induces a profound sensation of itch in humans.^58,61^ Potential pain-inducing effects of Compound 48/80 have not been studied extensively in humans, but Rukwied and coworkers show that Compound 48/80 and histamine, when applied to the forearm, induce similar sensations where itch is reported by all subjects and pain in 1 to 2 subjects.^53^ Therefore, we consider the behavioral response that subcutaneous injections of Compound 48/80 induces in the calf, primarily as an indicator of itch, although sensations of pain on an individual level cannot be excluded. Chemogenetic silencing attenuated the licking/biting response to Compound 48/80 in terms of duration and frequency compared with control mice (duration *P* = 0.0036, frequency *P* = 0.0029, Fig. 6D-E), indicating that the lumbar spinal Ucn3-Cre population contributes to the communication of Compound 48/80-evoked behavior. The role of the lumbar Ucn3-Cre population in mechanical and noxious heat transmission was examined using the von Frey and Hargreaves tests, respectively. Activation of the hM4Di receptor did not affect the withdrawal response to mechanical stimuli (*P* = 0.1002, Fig. 6F) or noxious heat (*P* = 0.3488, Fig. 6G), indicating that the spinal lumbar Ucn3-Cre population is not involved in the transmission of light touch nor noxious heat.

## 4. Discussion

In this study, we show that the Ucn3-Cre line marks an excitatory neuronal dorsal horn population that expresses *Ucn3* and can be divided into 2 nonoverlapping subpopulations expressing the mechanically related markers PKCγ or CALB2. Chemogenetic activation of lumbar Ucn3-Cre neurons evokes a targeted biting/licking behavior of the corresponding dermatome, and chemogenetic inhibition attenuates Compound 48/80-induced behavior. Furthermore, subpopulations of lumbar spinal *Ucn3* neurons express *fos* in response to scratching or Compound 48/80, further linking the lumbar Ucn3 neurons to mechanosensation and Compound 48/80-induced sensations. In line with these findings, lumbar Ucn3-Cre neurons were shown to receive monosynaptic input from primarily Aβ and C primary afferents.

### 4.1. Urocortin 3 is expressed in lumbar subpopulations involved in chemical itch and mechanosensation

The GENSAT project originally generated 2 different Ucn3-Cre founder lines: KF31 and KF43. Another study characterized the KF43 Ucn3-Cre line,^45^ whereas we have used the KF31 Ucn3-Cre line, and the lines target slightly different neuronal populations. Both studies show that spinal Ucn3-Cre cells are excitatory; however, Pan et al. detected PKCγ expression in only 25.1% and *Calb2* in 15.6% of spinal Ucn3-Cre neurons,^45^ indicating that almost 60% of the spinal Ucn3-Cre neurons were negative for PKCγ and CALB2. By contrast, we found that 97% of lumbar spinal Ucn3-Cre neurons express either of these markers; thus, the population studied by Pan et al. may include a large subset of cells that are not targeted in this study and vice versa. Furthermore, the reported differences regarding the overlap of Ucn3-Cre with the markers PKCγ and CALB2 can be due to differences in detection methods and antibody concentrations. Pan et al.^45^ used in situ hybridization to detect *Calb2* expression while we used immunofluorescence.

Spinal expression of PKCγ and CALB2 mark 2 largely nonoverlapping populations.^47^ Very weak expression of PKCγ has been reported in a subset of CALB2 expressing cells,^21^ in line with data from the Zeisel et al. dataset,^68^ where we identified *Calb2* expression in the population of cells with a low expression of *Prkcg*, but very little *Calb2* in the population with high *Prkcg* expression (Fig. S6A, http://links.lww.com/PAIN/C154). Low PKCγ-expressing, CALB2-positive cells were not targeted by a PKCγ-CreERT2 reporter^47^ and were not detected in the present immunohistology study, possibly due to differences in detection sensitivity. Instead, our immunostaining confirms the separation of the CALB2 and PKCγ populations and indicates that the lumbar spinal *Ucn3* population defined by the KF31 Ucn3-Cre line covers parts of these 2. Earlier work on PKCγ and CALB2 populations has shown specific roles in pain behaviors to nonoverlapping mechanical stimuli. Depletion of PKCγ, or selective silencing of the PKCγ population, does not affect inflammation-induced mechanical allodynia but decreases mechanical hypersensitivity after nerve injury.^38,47^ Silencing of lumbar Calb2-Cre neurons instead reduces the responses to mechanical stimuli after inflammation, while neuropathic mechanical allodynia remains unaffected.^47^ In addition, mice in which Lbx1-Flpo; Calb2-Cre neurons were ablated using diphtheria toxin in adulthood displayed decreased sensitivity to mechanical stimuli by von Frey filaments.^12^

Analysis of the KF43 Ucn3-Cre line shows that ablation of spinal Ucn3-Cre neurons does not alter responses to von Frey stimulation targeting the paw in the baseline condition.^45^ Instead, ablation of spinal Ucn3-Cre neurons resulted in reduced scratching upon mechanical stimulation in dry skin conditions, histamine alloknesis, and allergic contact dermatitis, as well as behind the ears in the baseline condition, whereas the scratching behavior after Compound 48/80-, chloroquine-, Me-5-HT-, or SLIGRL-injection was unaffected.^45^ Thus, the authors concluded that spinal Ucn3-Cre neurons labeled by the KF43 line transmit mechanical itch. Worthy to note is that the authors could not detect *Ucn3* expression in the spinal cord in the KF43 Ucn3-Cre line; they did however detect *Ucn3* expression in Ucn3-Cre neurons in the hypothalamus.^45^ Our analysis of the KF31 Ucn3-Cre line showed that the lumbar Ucn3-Cre neurons express *Ucn3* to a high extent. In addition, our analysis demonstrated that the Ucn3-Cre population can be divided into 2 nonoverlapping populations that express either PKCγ or CALB2, which is consistent with the single-cell RNA sequencing data of the spinal *Ucn3* population.^68^ Chemogenetic inhibition of the lumbar Ucn3-Cre population did not alter mechanical von Frey thresholds or the withdrawal latency to noxious heat. Instead, the biting/licking behavior induced by Compound 48/80 was reduced with respect to both frequency and duration, associating lumbar *Ucn3* neurons to the communication of Compound 48/80-induced sensations. Analysis of lumbar *fos* expression supports this finding as a subpopulation of lumbar *Ucn3* neurons expressed *fos* upon injection of Compound 48/80 in the corresponding dermatome. Compound 48/80 degranulates mast cells and induces a profound sensation of itch in humans.^58,61^ In addition to the Compound 48/80 phenotype, our *fos* analysis revealed that a subpopulation of lumbar *Ucn3* neurons was activated by artificial scratching. Several studies have mapped *fos* expression patterns in the dorsal horn after different peripheral stimulations, where the mediolateral location of activated cells depends on the location of the stimulus in relation to the somatotopic organization of the dorsal horn.^8,29,57^ Pruritic compounds and subsequent scratching predominantly activate neurons in the superficial lateral dorsal horn,^17,65^ while noxious and nonnoxious stimuli activate superficial to deep neurons in the dorsal horn.^17^ Our *fos* analysis showed that Compound 48/80 evoked *fos* promotor activity in the lateral dorsal horn, in line with the pattern observed in previous analyses using pruritogens,^17,65^ and that more *Ucn3* neurons were activated on the ipsilateral side compared with the saline-injected control mice. A few studies have investigated the *fos* pattern caused by scratching,^17,67^ but these have been in combination with itch-inducing compounds. Hence, our study provides a unique insight into the spinal neurons that are activated by the scratching with no possible confounding factor from the itch sensation. Future studies, for instance using single-cell mRNA sequencing analysis, could investigate the molecular identity of the *fos/Ucn3* cells further to elucidate whether they are activated by several sensory stimuli and/or if single-modality *Ucn3* subpopulations exist. Scratching behavior targeting the calf, using the paw, is not a natural behavior for mice. We chose this model because the paw is used for scratching hairy skin on most parts of the body, making it a relevant model to investigate the effects of scratching, and because of the difficulty of artificially mimicking the biting behavior.

We noticed *Ucn3* mRNA also in deeper regions of the dorsal horn, which is slightly different from the Cre activity detected in the reporter line or using viral reporters, where the expression of Ucn3-Cre is mainly found in the more superficial layers. An analysis of the *tdTomato* and *Ucn3* mRNA patterns revealed that both *tdTomato* and *Ucn3* can be detected in deeper lamina compared with the Cre activity. This discrepancy may be due to the regulation of expression on a translational level, as RNAscope is a sensitive method that can detect a low number of mRNA molecules that may not be translated into protein. The *Ucn3* analyses detected expression levels between 71% and 76% in tdTomato or virally labeled Ucn3-Cre cells, which could be due to the variable and sporadic mRNA expression in individual cells.^6,31,60^

### 4.2. Lumbar spinal Urocortin 3 neurons receive monosynaptic input from Aβ and C dorsal root ganglia neurons

Itchy stimuli are detected in the skin by peripheral C fibers,^55^ and mechanical stimuli are detected by mechanically sensitive low threshold mechanoreceptors (LTMRs) and high threshold mechanoreceptors (HTMRs).^41^ We characterized the synaptic input to lumbar Ucn3-Cre neurons and found that a large proportion (84%) of the input from primary afferents consists of myelinated NF200(+) fibers. Myelinated fibers belong to Aα proprioceptive, Aβ LTMRs, Aβ HTMRs, and Aδ neurons.^11,41,43^ As only 1% of the traced neurons coexpressed the Aα marker PARV and 21% displayed overlap with the nociceptive marker TRKA, substantial inputs from Aβ LTMRs and/or Aβ HTMRs are plausible. Indeed, our patch-clamp recordings with dorsal root activation confirmed that lumbar spinal Ucn3-Cre neurons received monosynaptic inputs from Aβ fibers. Moreover, our tracing and electrophysiological analyses showed that 8% of the traced DRG neurons expressed CGRP and that spinal Ucn3-Cre neurons received monosynaptic inputs from C fibers, respectively. No overlap was detected with a marker for C-LTMRs, TH. There is, however, evidence that nonpeptidergic unmyelinated populations, including the TH population, may be resistant to retrograde infection by rabies virus,^2^ and hence, we cannot exclude innervation by C-LTMRs. In summary, these results indicate that the lumbar Ucn3-Cre neurons receive peripheral input from both mechanically sensitive peripheral fibers and C fibers.

In conclusion, the lumbar spinal *Ucn3* population expresses markers associated with mechanical sensation, and a subpopulation is activated by scratching. The population receives peripheral input from both touch- and itch/pain-associated fiber types and is under local tonic inhibition by primarily glycinergic transmission. Furthermore, the lumbar spinal *Ucn3* population is activated by Compound 48/80, and consequently, chemogenetic inhibition of lumbar Ucn3-Cre neurons reduced biting and licking induced by Compound 48/80. Future analyses could address how lumbar *Ucn3* neurons are affected by peripheral sensitization induced by nerve injury, inflammation, or allergic conditions to increase the understanding of these nerve cells in pathological conditions.

## Conflict of interest statement

The authors have no conflicts of interest to declare.

## Appendix A. Supplemental digital content

Supplemental digital content associated with this article can be found online at http://links.lww.com/PAIN/C154.
