# Supplementary material for: Spinal lumbar Urocortin 3-expressing neurons are associated with both scratching and Compound 48/80-induced sensations
Source: Pain. 2024 Oct 15;166(5):1070–87. doi: 10.1097/j.pain.0000000000003435 (PMC12004988; doi:10.1097/j.pain.0000000000003435)
Supplement: Supplementary file 1 [file jop-166-1070-s001.pdf]

Supplementary information

Spinal lumbar Urocortin3-expressing neurons are associated with both scratching and Compound 48/80-induced sensations

Marina C.M. Franck, Hannah M. Weman, Mikaela M. Ceder, Aikeremu Ahemaiti, Katharina Henriksson, Erica Bengtsson, Kajsa A. Magnusson, Harmen K. Koning, Caroline Öhman-Mägi, Malin C. Lagerström

Number of pages: 14

Number of figures: 7

Number of file references: 2

Number of tables: 1

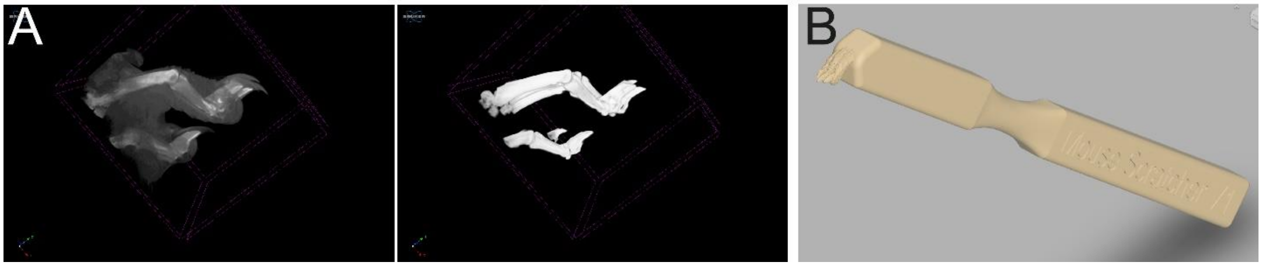

Figure S1. 3D printing of artificial mouse claw in “scratching position”, relates to Figure 4.

A. CT-scan of a mouse claw in “scratching position” with tissue and bones (left) and only bones (right). **B.** The finalized 3D printed product, “Mouse Scratcher V1” based on (A).

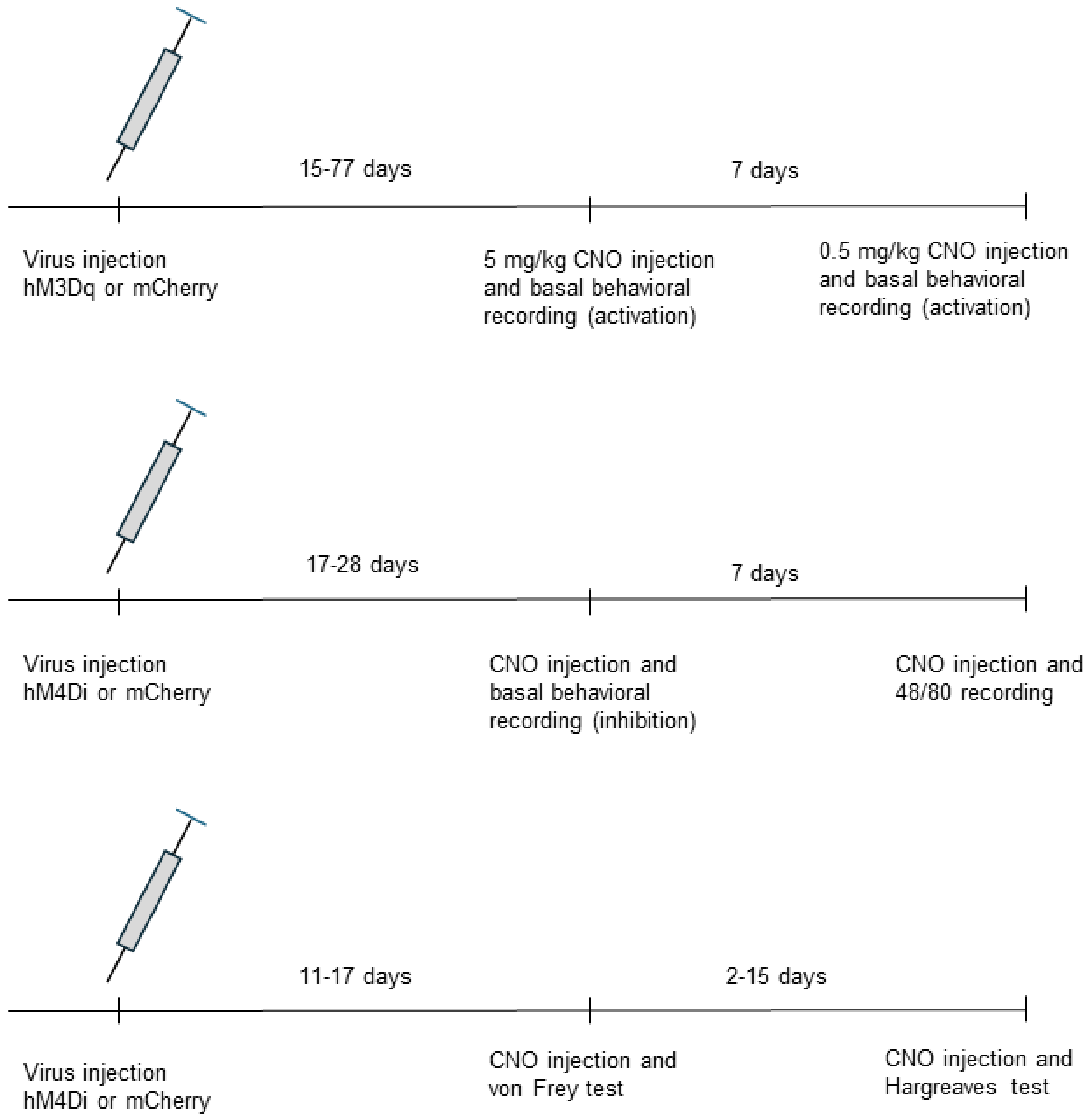

Figure S2. Experimental groups and protocols for behavioral experiments, relates to Figure 6. Animals were subjected to one viral injection and two behavioral experiments in the order illustrated.

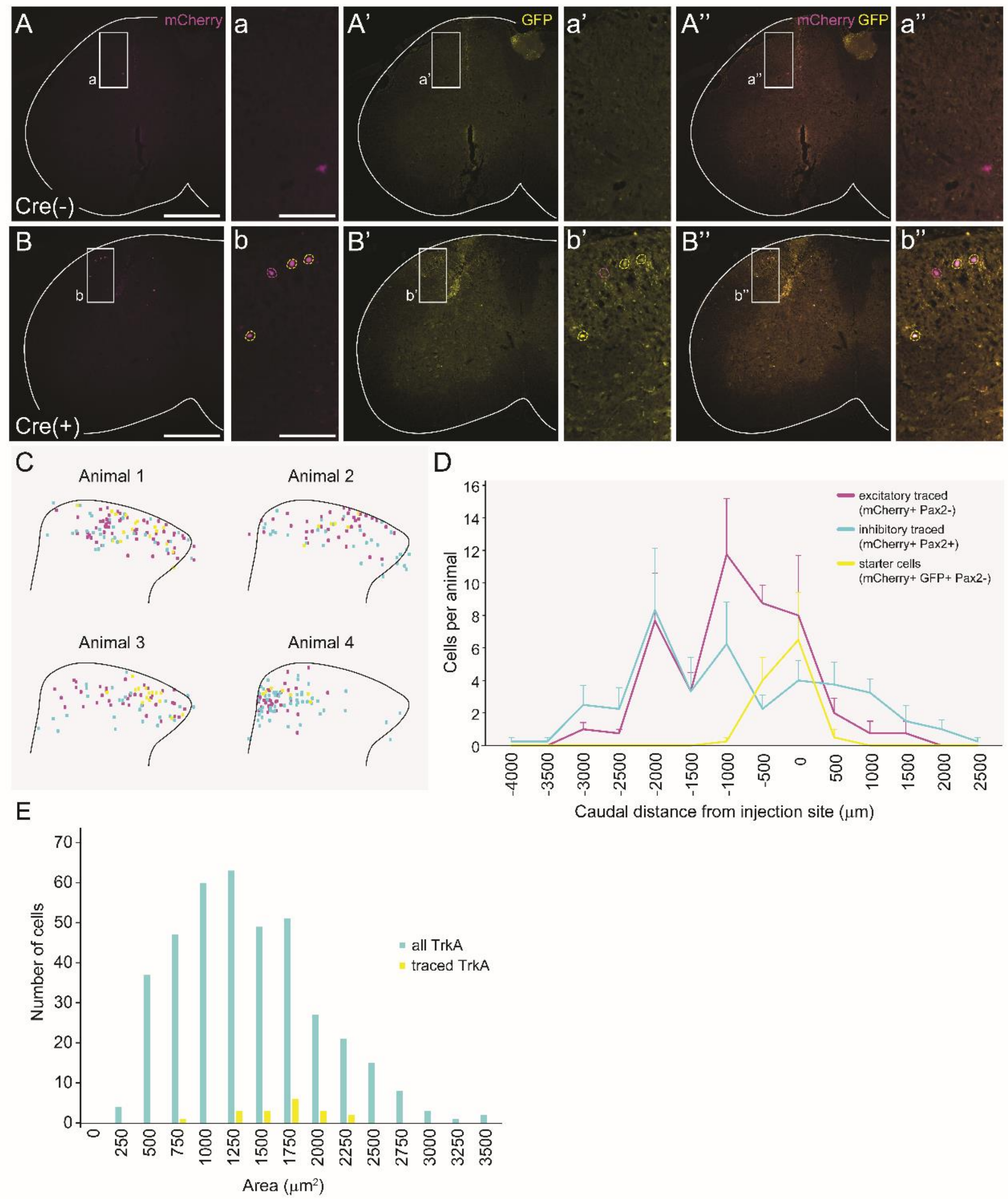

Figure S3. Retrograde rabies virus-based trans-synaptic tracing, relates to Figure 2. A–B. Starter cells (mCherry(+)GFP(+)) are not found in Cre(-) controls (A, n = 2), only in Cre(+) traced mice (B, n = 4). Yellow dashed lines indicate mCherry(+)GFP(+) starter cells and magenta dashed lines show a mCherry(+)GFP(-) traced cell. **C.** Locations of inhibitory (PAX2(+)) (cyan) traced cells, excitatory (PAX2(-)) (magenta) traced cells and starter cells (yellow) in the ipsilateral dorsal horn of the four Ucn3-Cre mice used in the analysis. **D.** Distribution of PAX2(-)

(magenta) traced cells, PAX2(+) (cyan) and starter cells (yellow) along the rostrocaudal axis of the ipsilateral dorsal horn. **E.** Size distribution of all TRKA(+) DRG cells and traced TRKA(+) DRG cells. Scale bar in A-B: 1 mm, 250 μm for enlargement.

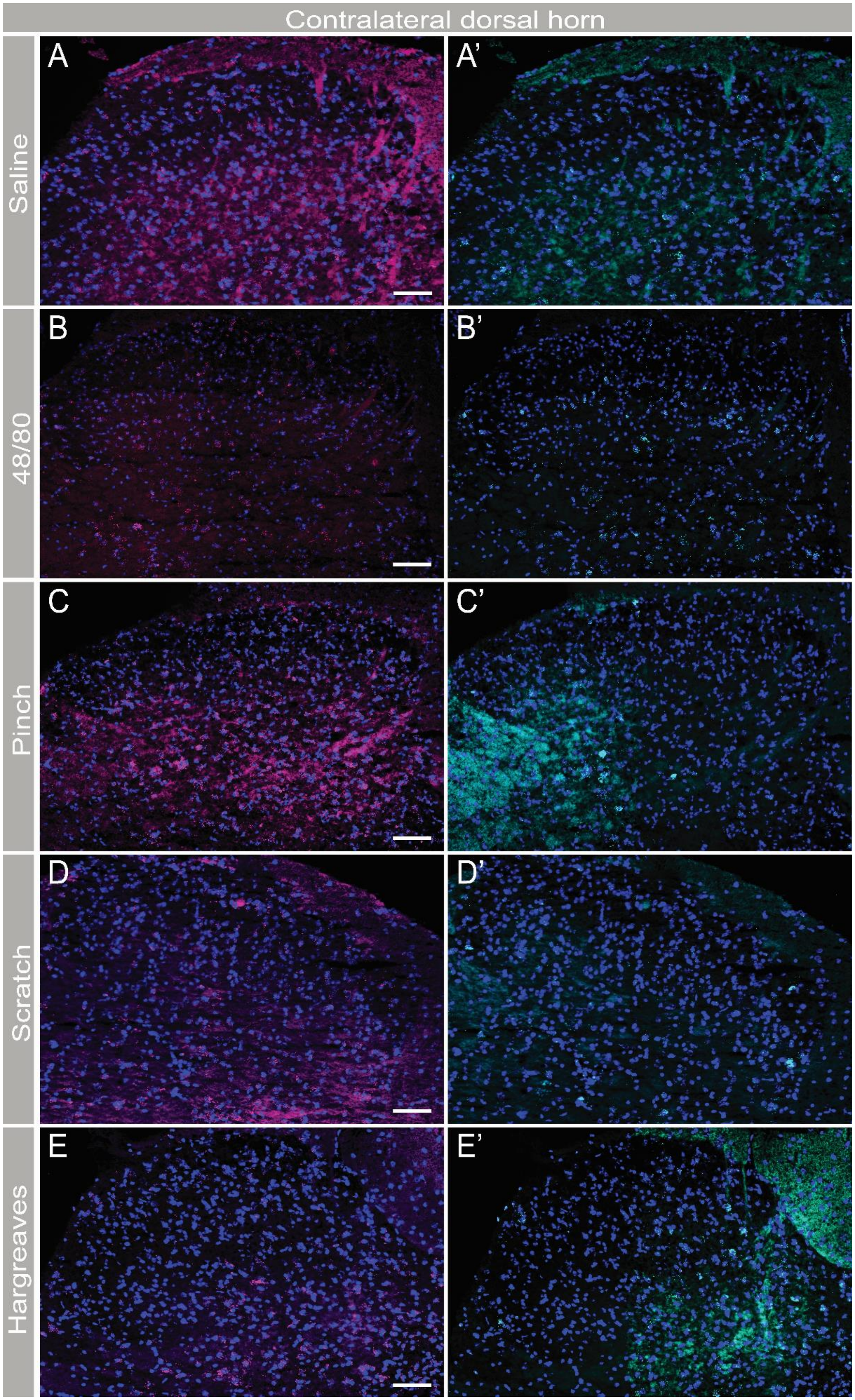

Contralateral dorsal horn
Saline
A
A'
48/80
B
B'
Pinch
C
C'
Scratch
D
D'
Hargreaves
E
E'

Figure S4. ***Ucn3*** **and** ***fos*** **probe pattern in the contralateral dorsal horn, relates to Figure 4.** Showing the separate channels for *Ucn3* (magenta), *fos* (cyan) and DAPI (dark blue) on the contralateral dorsal horn for (**A–A'**) saline, (**B–B'**) Compound 48/80, (**C–C'**) pinch, (**D–D'**) artificial scratching, and (**E–E'**) noxious heat (Hargreaves). Scale bars: 100 µm.

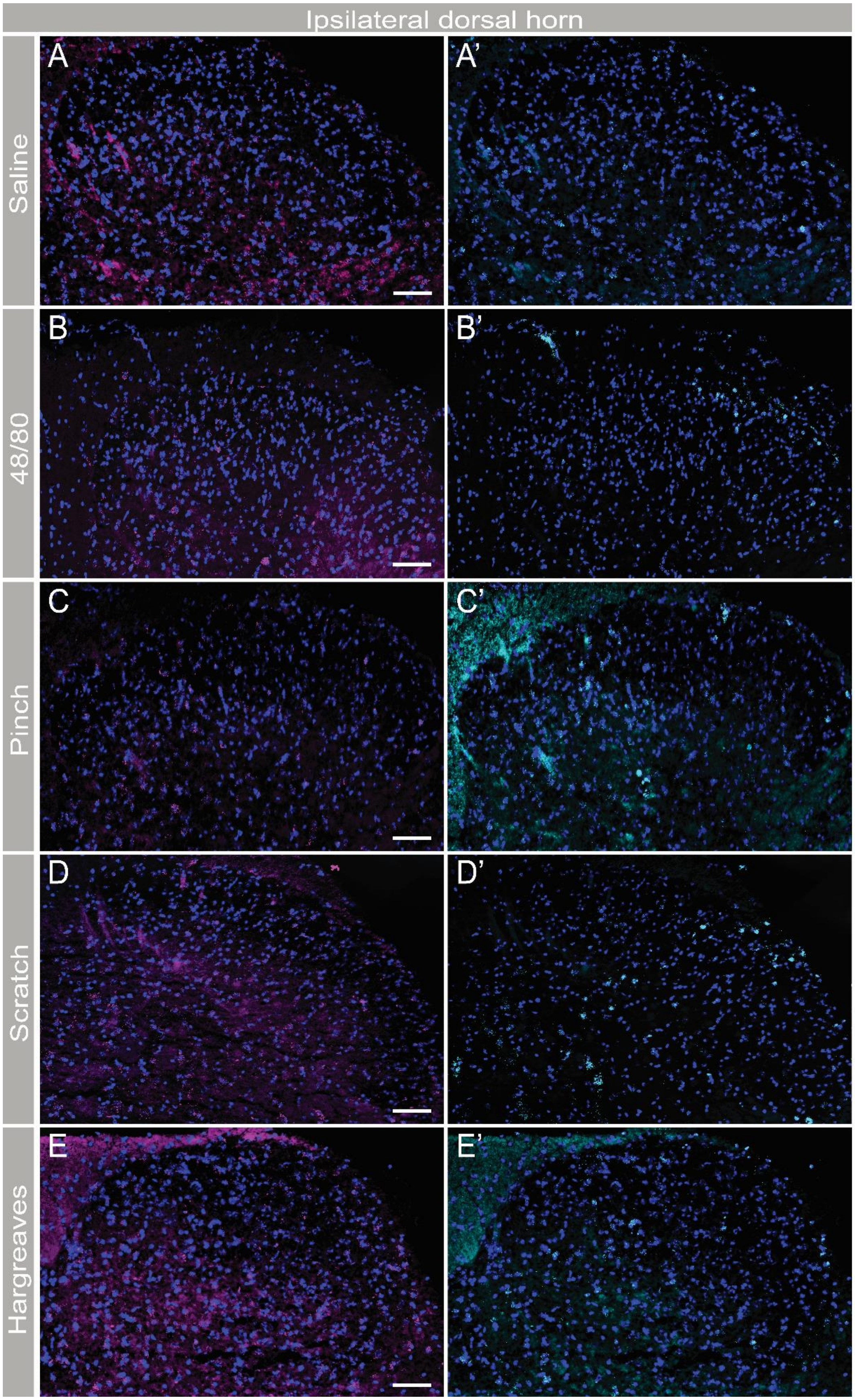
Ipsilateral dorsal horn
Saline
48/80
Pinch
Scratch
Hargreaves
A
A'
B
B'
C
C'
D
D'
E
E'

Figure S5. ***Ucn3*** **and** ***fos*** **probe pattern in the ipsilateral dorsal horn, relates to Figure 4.** Showing the separate channels for *Ucn3* (magenta), *fos* (cyan) and DAPI (dark blue) on the ipsilateral dorsal horn for (**A–A'**) saline, (**B–B'**) Compound 48/80, (**C–C'**) pinch, (**D–D'**) artificial scratching, and (**E–E'**) noxious heat (Hargreaves). Scale bars 100 μm.

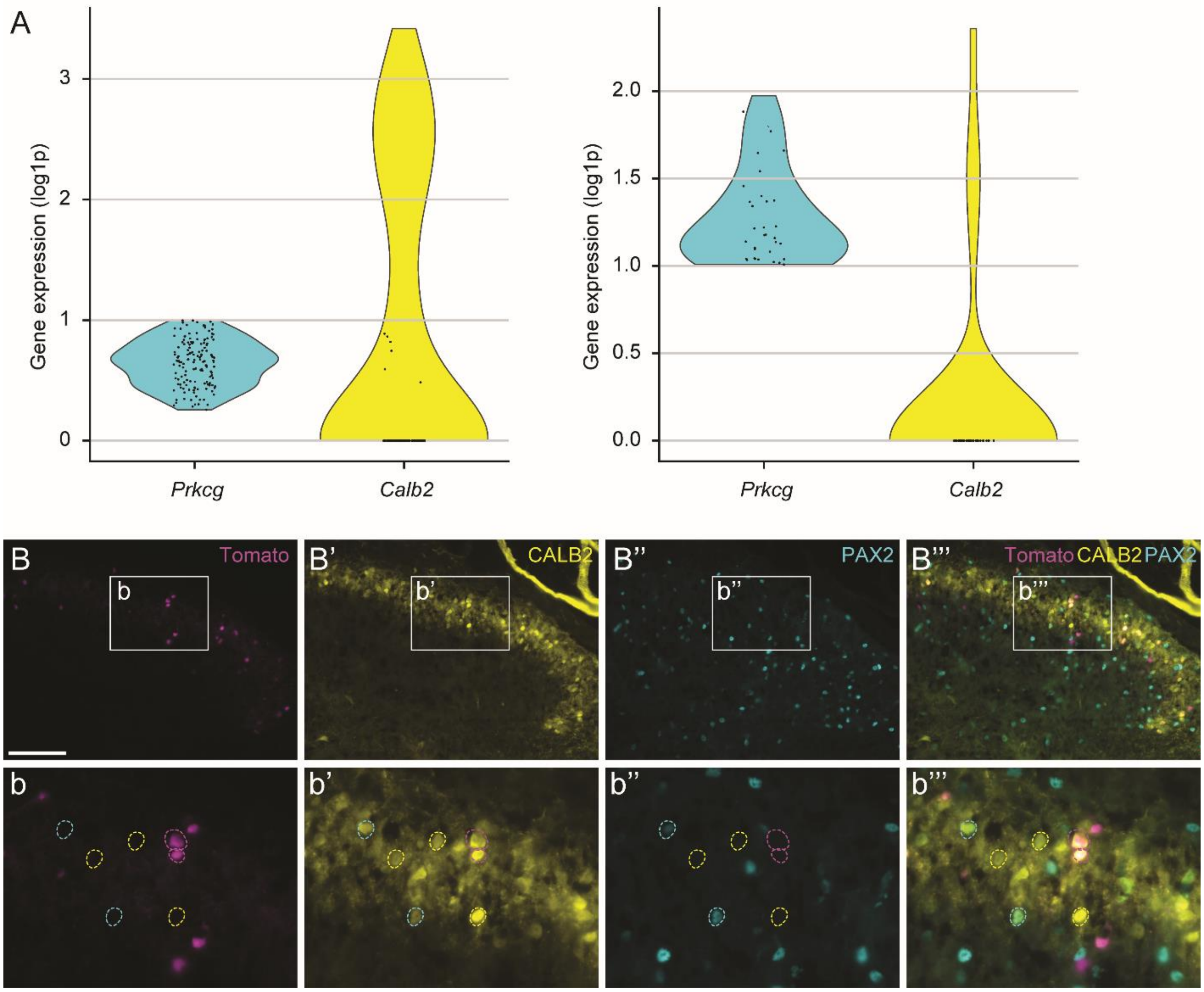

Figure S6. Ucn3-Cre;tdTomato is expressed in a subset of CALB2(+)/PAX2(-) neurons, relates to Figure 5. **(A)** Single-cell RNA analysis of *Calb2* expression in low *Prkcg*-expressing (0.1 > *Prkcg* log1p > 1.0, left) and high *Prkcg*-expressing (*Prkcg* log1p > 1.0, right) cells in the Zeisel et al. dataset. **(B–B''')** Immunohistochemical analysis of CALB2(+) cells in the spinal Ucn3-Cre;tdTomato population. Cyan dashed lines in magnifications **b–b'''** denote cells co-expressing CALB2 and the inhibitory marker PAX2, yellow dashed lines show CALB2(+)PAX2(-) and magenta colored dashed lines show CALB2(+)PAX2(-) Ucn3-Cre;tdTomato cells. Scale bar 200 μm, for magnifications 67 μm.

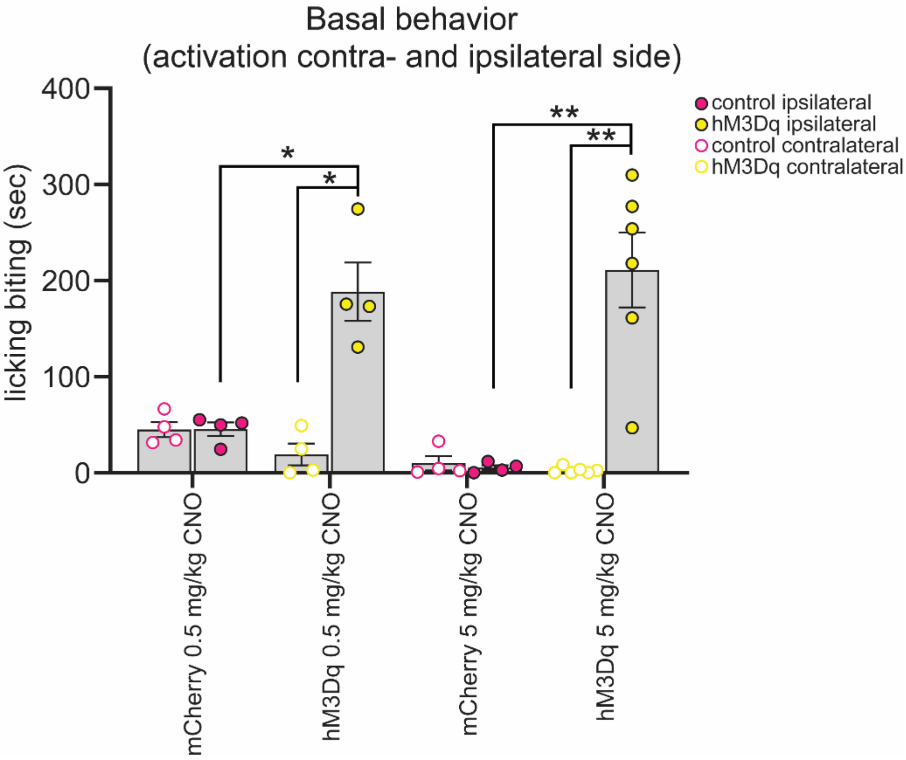

Figure S7. Licking and biting behavior towards the contralateral and ipsilateral hind paw in control mice and mice with chemogenetic activation of the lumbar spinal Ucn3-Cre neurons, relates to Figure 6. Activation of Ucn3-Cre neurons using viral expression of AAV8/hSyn-DIO-hM3Dq-mCherry and administration of CNO caused a dose-dependent biting/licking behavior (Kruskal-Wallis p=.0002, Mann-Whitney post-hoc test 0.5 mg/kg CNO control ipsilateral vs hM3Dq ipsilateral p=.0286, hM3Dq contralateral vs hM3Dq ipsilateral p=.0286; 5 mg/kg CNO control ipsilateral vs hM3Dq ipsilateral p=.0095, hM3Dq contralateral vs hM3Dq ipsilateral p=.0022) towards the ipsilateral paw (corresponding dermatome) compared with the control virus without the hM3Dq receptor. No significant behavioral difference was seen on the contralateral side (0.5 mg/kg CNO p=.200; 5 mg/ml CNO p=.2610). Note that the data points from the ipsilateral side are the same as in Figure 6.

Supplementary File 1. The STL file of the "Mouse scratcher V1". This STL file describes the surface geometry of the artificial mouse scratcher "Mouse Scratcher V1" (MouseScratcher_v1.stl).

Supplementary File 2. The FORM file of the "Mouse scratcher V1". The FORM file describes the print settings of the STL file to enable the 3D printing of the artificial mouse scratcher (MouseScratcher_vi_formfile.form).

Table S1. The top differentially expressed genes in the *Ucn3* expressing SCGLU7 and SCGLU9 clusters in the Zeisel et al. dataset [1]**.** The top differential gene expression in comparison to each other. FDR = false discovery rate.

SCGLU7			SCGLU9		
Gene	z-score	FDR	Gene	z-score	FDR
Chgb	8.48	1.27×10^{-4}	*Trh*	11.81	8.77×10^{-13}
Calb2	7.97	7.45×10^{-4}	*Snca*	9.26	5.48×10^{-9}
Nrxn1	7.25	4.00×10^{-6}	*Nts*	8.51	4.75×10^{-8}
Ly6h	7.16	7.00×10^{-6}	*Necab2*	8.12	2.64×10^{-7}
Cck	6.89	6.00×10^{-6}	*Pura*	7.85	3.17×10^{-7}
A230065H16Rik	5.66	1.34×10^{-2}	*Vat1l*	7.63	5.86×10^{-7}
Pcp4	5.66	8.29×10^{-4}	*Synpr*	7.54	3.27×10^{-7}
Phlda1	5.63	1.19×10^{-2}	*Car8*	7.25	1.81×10^{-6}
Laptm4b	5.56	1.48×10^{-2}	*Gpx3*	7.25	1.81×10^{-6}
H3f3b	5.22	2.70×10^{-3}	*Nr2f1*	7.24	1.23×10^{-6}
Mgp	5.09	2.67×10^{-2}	*Zbtb20*	7.10	6.91×10^{-6}
Nppc	5.03	2.73×10^{-2}	*Kcnip2*	6.83	6.46×10^{-6}
Ypel3	5.00	1.09×10^{-2}	*Tshz2*	6.77	6.46×10^{-6}
Lmo3	4.85	3.44×10^{-2}	*Calb1*	6.46	4.71×10^{-4}
Gm2694	4.85	1.47×10^{-2}	*Arpp21*	6.41	2.33×10^{-5}
Grin2b	4.80	2.67×10^{-2}	*Rmst*	6.40	1.63×10^{-4}
Kcnk1	4.78	3.38×10^{-2}	*Cmas*	6.16	6.34×10^{-5}
Serpine2	4.78	1.77×10^{-2}	*Prrxl1*	5.96	5.54×10^{-4}
Tpm1	4.29	5.00×10^{-2}	*Chst1*	5.82	1.38×10^{-4}
Cpne4	4.14	8.75×10^{-2}	*Rprm*	5.79	2.00×10^{-4}
Cdh8	4.02	9.60×10^{-2}	*Ift22*	5.77	1.45×10^{-4}

Tmsb4x	3.92	$6.32x\ 10^{-2}$	*Hspb1*	5.67	$2.99x\ 10^{-4}$
Rpl32	3.84	$4.63x\ 10^{-2}$	*Pde11e*	5.53	$4.71x\ 10^{-4}$
Cbln1	3.81	$8.98x\ 10^{-2}$	*Nrsn1*	5.43	$3.23x\ 10^{-3}$
Pde4d	3.57	0.16	*Tac1*	5.29	$9.88x\ 10^{-4}$
Trhde	3.52	0.17	*Tmeff2*	5.16	$1.55x\ 10^{-3}$
Cadm1	3.52	0.17	*Rasd1*	5.10	$2.18x\ 10^{-3}$
Nkain4	3.49	0.18	*Umad1*	5.07	$2.06x\ 10^{-3}$
Syt6	3.46	0.18	*Prrc2b*	5.04	$2.18x\ 10^{-3}$
Itm2c	3.45	0.15	*Tardbp*	4.91	$3.23x\ 10^{-3}$
Dpcd	3.41	0.15	*Rcan2*	4.89	$3.23x\ 10^{-3}$
Rps26	3.37	0.17	*Got1*	4.87	$3.23x\ 10^{-3}$
Auts2	3.33	0.20	*Esd*	4.86	$3.54x\ 10^{-3}$
Cntnap2	3.32	0.20	*Tspan5*	4.84	$3.60x\ 10^{-3}$
Spock3	3.29	0.20	*Pithd1*	4.83	$3.60x\ 10^{-3}$
Serp2	3.24	0.19	*Rbfox3*	4.83	$3.60x\ 10^{-3}$
Tmsb10	3.24	0.16	*Shisa8*	4.73	$4.98x\ 10^{-3}$
Cdh13	3.22	0.22	***Prkcg***	4.70	$4.98x\ 10^{-3}$
Ndufc2	3.15	0.20	*Ntrk3*	4.68	$5.84x\ 10^{-3}$
Gabra5	3.12	0.26	*Glo1*	4.65	$6.24x\ 10^{-3}$

[1] Zeisel A, Hochgerner H, Lonnerberg P, Johnsson A, Memic F, van der Zwan J, Haring M, Braun E, Borm LE, La Manno G, Codeluppi S, Furlan A, Lee K, Skene N, Harris KD, Hjerling-Leffler J, Arenas E, Ernfors P, Marklund U, Linnarsson S. Molecular Architecture of the Mouse Nervous System. Cell 2018;174(4):999-1014 e1022.
